# Effects of desiccation stress on adult female longevity in *Aedes aegypti* and *Ae. albopictus* (Diptera: Culicidae): results of a systematic review and pooled survival analysis

**DOI:** 10.1186/s13071-018-2808-6

**Published:** 2018-04-25

**Authors:** Chris A. Schmidt, Genevieve Comeau, Andrew J. Monaghan, Daniel J. Williamson, Kacey C. Ernst

**Affiliations:** 1 Department of Epidemiology and Biostatistics, Mel & Enid Zuckerman College of Public Health, University of Arizona, 1295 N. Martin Ave, Tucson, AZ 85724 USA; 2 National Center for Atmospheric Research, P.O. Box 3000, Boulder, CO 80307 USA; 3 Department of Entomology, College of Agriculture & Life Sciences, University of Arizona, P.O. Box 210036, Tucson, AZ 85721 USA

**Keywords:** *Aedes aegypti*, *Aedes albopictus*, Longevity, Survival, Humidity, Vapor pressure, Temperature, Review, Pooled analysis

## Abstract

**Background:**

Transmission dynamics of mosquito-borne viruses such as dengue, Zika and chikungunya are affected by the longevity of the adult female mosquito. Environmental conditions influence the survival of adult female *Aedes* mosquitoes, the primary vectors of these viruses. While the association of temperature with *Aedes* mortality has been relatively well-explored, the role of humidity is less established. The current study’s goals were to compile knowledge of the influence of humidity on adult survival in the important vector species *Aedes aegypti* and *Ae. albopictus*, and to quantify this relationship while accounting for the modifying effect of temperature.

**Methods:**

We performed a systematic literature review to identify studies reporting experimental results informing the relationships among temperature, humidity and adult survival in *Ae. aegypti* and *Ae. albopictus*. Using a novel simulation approach to harmonize disparate survival data, we conducted pooled survival analyses via stratified and mixed effects Cox regression to estimate temperature-dependent associations between humidity and mortality risk for these species across a broad range of temperatures and vapor pressure deficits.

**Results:**

After screening 1517 articles, 17 studies (one in semi-field and 16 in laboratory settings) met inclusion criteria and collectively reported results for 192 survival experiments. We review and synthesize relevant findings from these studies. Our stratified model estimated a strong temperature-dependent association of humidity with mortality in both species, though associations were not significant for *Ae. albopictus* in the mixed effects model. Lowest mortality risks were estimated around 27.5 °C and 21.5 °C for *Ae. aegypti* and *Ae. albopictus*, respectively, and mortality increased non-linearly with decreasing humidity. *Aedes aegypti* had a survival advantage relative to *Ae. albopictus* in the stratified model under most conditions, but species differences were not significant in the mixed effects model.

**Conclusions:**

Humidity is associated with mortality risk in adult female *Ae. aegypti* in controlled settings. Data are limited at low humidities, temperature extremes, and for *Ae. albopictus*, and further studies should be conducted to reduce model uncertainty in these contexts. Desiccation is likely an important factor in *Aedes* population dynamics and viral transmission in arid regions. Models of *Aedes*-borne virus transmission may be improved by more comprehensively representing humidity effects.

**Electronic supplementary material:**

The online version of this article (10.1186/s13071-018-2808-6) contains supplementary material, which is available to authorized users.

## Background

*Aedes* mosquitoes are vectors of multiple viruses of major public health significance, including dengue (DENV), yellow fever (YFV), chikungunya (CHIKV) and Zika (ZIKV) viruses [1]. Over the past four decades, *Aedes* (*Stegomyia*) *aegypti* (Linnaeus) and *Ae.* (*S.*) *albopictus* (Skuse) have expanded rapidly across the globe [2–4]. Both vectors are now well-established throughout much of the tropics and subtropics. Concurrently, DENV has continued to expand in the Western Hemisphere [5] and to cause a substantial global disease burden [6], CHIKV and ZIKV have emerged as significant threats in the Americas and elsewhere [7, 8], and recent outbreaks of YFV in central Africa and Brazil have increased concerns about its epidemic potential [9]. As continued transmission of *Aedes*-borne viruses is anticipated, proactive systems are needed to monitor and forecast times and areas of high transmission risk.

Process-based mathematical models of mosquito populations and arboviral transmission have enhanced the ability to forecast disease dynamics and predict impacts from vector control efforts [10–18]. Adult survival rate or longevity is a highly influential component of mosquito population models and vectorial capacity equations given its relationship to average generation time, fecundity and biting rate, and its interaction with viral extrinsic incubation periods (“EIP”; [18–22]). *Aedes* females newly infected with a viral pathogen must survive long enough for the virus to multiply and reach its salivary glands before the virus can be transmitted to a new host, and longer lifespans beyond the EIP increase the potential number of hosts that may be newly infected [22–24].

Environmental factors strongly influence the life-cycle and vectorial capacity of *Ae. aegypti* and *Ae. albopictus.* For example, temperature affects adult longevity, immature development, EIP, and other components of *Aedes* life history and vectorial capacity [18, 22, 25–32]. The association of temperature with adult survival frequently features in *Aedes* population and disease models (e.g. [10, 11, 14, 33]), and a recent study quantified this relationship to improve model parameterization [34]. Humidity also influences the ecological dynamics and life-cycle of *Aedes* mosquitoes in numerous ways, as reviewed below in reference to (i) the ecology and seasonality of *Aedes* and *Aedes*-transmitted viruses; (ii) desiccation of *Aedes*; and (iii) *Aedes* container habitats.

Numerous analyses have found humidity to be directly or indirectly associated with the ecology and seasonality of *Aedes* and with the incidence of associated viral diseases (e.g. [35–42]). Hales et al. [43], for example, identified annual average vapor pressure (a measure of humidity) as the best climatic predictor of the global distribution of dengue. Some studies have failed to find this relationship, however (e.g. [44]). *Aedes* abundance and *Aedes*-transmitted virus incidence show distinct seasonality in many regions globally, and may be indirectly linked to humidity through annual cycles of temperature and precipitation [31]. Seasonal increases in *Aedes* mosquito abundance and/or dengue incidence are associated with rainfall in Asia [45–48] and the Americas [49–52]. Rainfall is likely a driver of seasonality as it creates aquatic habitat for immature mosquito stages (e.g. [28, 53]), but is not a prerequisite for *Aedes* occurrence as water-filled containers are often manually filled throughout the year [54].

Humid conditions coincide with the moist air masses that bring rainfall [55], as warm season rainfall is driven by convective heating and occasional mechanical uplift, both very localized phenomena, but precipitation requires the generally widespread advection of moist unstable air [56]. For example, the North American monsoon is characterized by large-scale regional influxes of humidity which may not generate precipitation in a given time and place due to local topography and atmospheric dynamics [55, 57], but might be sufficient to moderate desiccation stress and evaporation rates. Conversely, relative humidity may also vary at small scales irrespective of precipitation, for example in relation to land use within urban environments [58] at scales that are likely to be relevant to urban mosquitoes such as *Ae. aegypti*. This partial decoupling of humidity and precipitation suggests that rainfall may be an incomplete proxy for humidity in *Aedes* models. More explicitly and completely quantifying the effects of humidity may improve model simulations of the ecology and seasonality of viral transmission by *Aedes*. Such improvements may be particularly impactful for simulations in understudied arid and semi-arid regions, where seasonal fluctuations of humidity can be substantial (e.g. [57]), and where 30% of the global population resides [59].

Humidity can impact *Aedes* mosquito survival directly through desiccation effects on eggs and adults (e.g. [60]), with the rate of water loss in adult mosquitoes increasing with decreasing humidity [61]. Desiccation (extreme drying) affects the volume and osmolarity of hemolymph in the hemocoel of adult insects [62]. Normal water content in adult insects ranges from 40–90% of wet mass [62], and *Aedes* mosquitoes can lose up to 40% of their water content before dying [63]. Process-based models have endeavored to include the desiccation effects of humidity. For example, Lega et al. [64] report that inclusion of humidity into life-cycle models improves model fit to field data, despite their study using a blunt approach, with a one-step increase in adult survival between 72% and 95% relative humidity. Similarly, the CIMSiM and Skeeter Buster models [10, 11, 20, 65] use a simple three-step function to define the relationship between humidity and adult survival. However, the absence of a comprehensive review and quantitative model of humidity-associated adult mortality in *Aedes* has hindered its full incorporation into ecological and epidemiological models [18, 34].

Humidity may also indirectly affect *Aedes* populations by modulating evaporation rates from water-filled containers in which larvae and pupae develop (e.g. [66–68]). Fluctuations in humidity can have striking effects on evaporation rates. For a given temperature, the evaporation rate of water is about four times larger when relative humidity (RH) is 40 *vs* 85%. The former value is characteristic of arid cities such as Phoenix, Arizona, while the latter value is common in humid subtropical cities such as Miami, Florida. Additionally, as temperatures incrementally increase, evaporation rates increase non-linearly, meaning that rates are disproportionately larger at hotter temperatures (e.g. > 30 °C *vs* < 20 °C). Experimental studies have demonstrated that increased larval competition and reduced volume of larval development sites (i.e. via drying) can decrease the size of *Aedes* females at eclosion [69–71] and that body size likewise influences vectorial capacity [71–73].

In summary, further investigation into the role of humidity in the survival of *Aedes* mosquitoes and subsequent risk for *Aedes*-transmitted viruses is motivated by incomplete knowledge of the association of humidity with *Aedes* ecology and seasonality, the physiological effects of desiccation in adult mosquitoes, and the large fluctuations of evaporation and desiccation that can arise from regularly-observed changes in humidity. The objectives of the current study were to (i) compile knowledge of the influence of humidity on adult survival in *Ae. aegypti* and *Ae. albopictus* via a systematic review of published research, and (ii) assimilate this information in a pooled analysis to quantify the relationship between humidity and adult survival while accounting for the modifying effect of temperature. Our approach provides an opportunity to improve *Aedes* population and disease transmission models by accommodating a non-linear and temperature-dependent relationship between humidity and survival.

## Methods

Reporting follows the PRISMA (Preferred Reporting Items for Systematic Reviews and Meta-Analyses) statement [74].

### Literature search

Studies were identified by searching electronic databases without restrictions on language or publication year. Searches were conducted from 7–13 February 2016 in Web of Science (all databases), PubMed, Google Scholar, WHOLIS, Scopus, LILACS, PAHO, CUMED, and MEDCarib. Spanish translations of key terms were added to searches in the latter four databases. Search strings consisted of thematic terms related to *Aedes*, humidity, temperature, and longevity, and were structured for each database as necessitated by their specific formats. The full list of search strings is provided in Additional file 1. All results were downloaded and concatenated, except that only the top 200 hits (sorted by relevance) were retained from Google Scholar.

Abstracts of all unique articles were assessed independently by two authors (CS, GC) and retained for further review if they appeared to report results of experimental or observational studies of adult longevity in *Ae. aegypti* or *Ae. albopictus*. Database searches were supplemented by three rounds of forward and backward reference searches of candidate articles. Full text was retrieved for all articles considered potentially eligible by at least one reviewer, and was independently reviewed by the same two authors. Disagreements over final study inclusion were resolved by consensus with a third author (KE), and non-English language articles were evaluated with translation support. Articles were eligible for final inclusion in the review if they were formally published, reported original survival data for adult female *Ae. aegypti* or *Ae. albopictus* in laboratory, semi-field or field conditions, and met additional inclusion criteria as follows. Longevity data were required to be reported as raw individual survival times, survival curves, or mean or median longevities, with sample sizes indicated. Studies were required to report results from at least two cohorts exposed to different temperature-humidity regimens. While studies reporting experimental results from only a single temperature and humidity regimen may provide useful information about absolute mortality rates, the goal of our study was to estimate temperature and humidity effects on relative mortality, which requires the availability of two or more temperature-humidity regimes in a given study to establish a contrast. Studies were required to report experimental designs in sufficient detail to determine temperature, humidity, and water and nutritional provisioning, which a priori were considered essential influences on longevity, with other conditions kept constant among experiments. Mosquito cohorts were required to be unexposed to chemical treatment or intentional microbial infection (including *Wolbachia*) and to represent non-transgenic lineages; studies failing these criteria were examined for the presence of control groups that were otherwise eligible.

### Data extraction

**Fig. 1:**
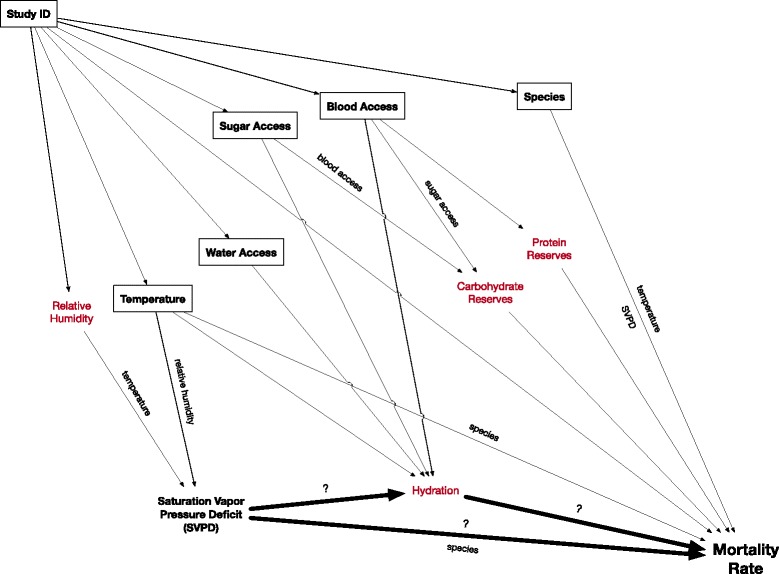
Directed acyclic graph (DAG) of hypothesized relationships among modeled exposure and outcome variables. Some hypothesized mediators (red text) were not directly modeled but are included here for explicatory purposes. Boxes reflect conditioning on variables in the statistical models to address potential confounding and colliding bias in estimating the association between SVPD and mortality, with variable names along paths reflecting modeled interactions

A directed acyclic graph (DAG) was developed to explicitly highlight hypothesized relationships among variables and support development of appropriate statistical models (Fig. 1). Key variables were extracted for mosquito cohorts in each study: (i) setting (laboratory, semi-field or field); (ii) species; (iii) total sample size across replicates; (iv) longevity (see below); (v) mean temperature and relative humidity; and (vi) separate variables for water, sugar and blood meal provision. As water, sugar and blood meals were provided using diverse methods and at widely varying frequencies, durations, and qualities, they were simplified to binary presence/absence variables indicating provision at any point after the start of an experiment. Water provisioning was considered present if a sugar solution or blood meal was provided, as these nutrition sources also provide hydration.

In cases where only temperature or humidity ranges were provided, the midpoints were taken to represent mean conditions. To analyze the role of a biologically meaningful measure of desiccation stress, saturation vapor pressure deficit (SVPD) was calculated from reported mean temperatures and relative humidities [75]. Saturation vapor pressure deficit is the difference between the actual vapor pressure in an air mass and the vapor pressure that would exist at saturation, which is non-linearly related to temperature. Depending on the format in which they were reported, survival data were extracted as (i) raw survival times for individual mosquitoes; (ii) mean or median longevities in days; or (iii) survival proportions at specified time points. Survival data were obtained from tables or via extraction from graphical survival curves or plots using GetData Graph Visualizer (v11.32521.0). Primary survival time data were requested via email from authors of most eligible studies published after 1995. To help assess possible climatic adaptations of *Aedes* populations used in each study reporting sufficient mosquito collection details, Köppen-Geiger climate classifications [76] of geographical localities of origin were estimated using ArcMap 10.2.2.

### Survival time simulations

All statistical analyses were conducted in R v3.3.1 [77]. We designed a simulation and pooled analysis approach which could flexibly harmonize disparate reporting formats among studies and model non-linear covariate-outcome relationships across the range of study conditions. Our strategy differs from a traditional meta-analysis in its use of simulated and reported data at the level of individual mosquitoes, rather than direct analysis of summary statistics or analytical results. For this reason, we refer to our study as a “pooled analysis” rather than a “meta-analysis”. Individual mosquito survival times were simulated from reported survival curves or longevity summaries for each mosquito cohort for which raw observed survival times were unavailable. Simulations were performed with the R packages *nls2* and *Runuran* [78, 79] under the Weibull and log-logistic distributions [80, 81]. Both models are commonly employed in survival analyses and accommodate age-varying mortality rates. The Weibull distribution has the property of proportional hazards, while the log-logistic is a proportional odds model [82].

Two-parameter Weibull and log-logistic cumulative distribution functions were fit via nonlinear least squares to observed survival curves for each experimental cohort that had a reported survival curve. A log-logistic or Weibull distribution was then selected with probability equal to the relative inverse residual sum of squares (RSS) of respective model fits for that cohort. Individual mosquito survival times were simulated using parameter estimates from the selected model. The number of simulated individual survival times for a cohort was equal to its reported sample size, and was distributed in proportion to the mortality events between each pair of reported observations (with rounding to the nearest integer). Simulated survival times were therefore internally calibrated by the reported survival proportions, with the fitted models guiding the distribution of survival times between observations. Right-censoring was modeled for experiments without complete follow-up, with censoring at the last observation time. Some cohorts required further simulation of additional survival times randomly drawn from the follow-up period, or random exclusion of excess individual times, to achieve the full sample size.

For cohorts with only a single censored survival observation or only a mean or median survival time, a Weibull or log-logistic model was randomly selected with probability equal to the relative inverse of summed RSS for all Weibull and log-logistic fitted models across experiments. Parameter values were drawn from prediction intervals of linear regression models relating estimated times of specified survival proportions to model scale or shape parameters among all fitted models. Survival proportions were either the proportion surviving at single censored observations, 0.5 for median survival times, or calculated survival proportions at mean survival times based on the properties of the Weibull and log-logistic distributions [80, 81]. A total of 500 replicate simulation runs were performed.

### Cox regression analysis

Simulated mosquito survival times were pooled across studies and analyzed using both stratified and mixed effects Cox regression models (*rms* and *coxme* R packages; [83, 84]). The stratified model (with stratification by study) allows studies to differ in their baseline hazard functions but to contribute to the pooled estimation of hazard ratios for modeled covariates [85]. Here the hazard ratio represents the relative risk of death (over very short timeframes) for mosquitoes under two contrasting sets of conditions, for example at a given value of temperature and/or humidity *vs* a reference value of temperature and/or humidity. The mixed effects model was identical to the stratified model but with the inclusion of study-level random effects instead of study-level stratification. We consider the stratified model to be the more appropriate analytical approach because it does not require an unrealistic assumption of identical baseline hazard functions across studies, but we included the mixed effects model as a check on the robustness of outputs from the stratified model.

The most appropriate humidity variable was determined via comparison of models containing relative humidity, vapor pressure or SVPD as covariates; SVPD yielded the model with the lowest Akaike information criterion (AIC) score and was selected for all remaining analyses. Covariates in the final models included temperature, SVPD, and water, sugar and blood provisioning. From a survival standpoint, sugar and blood meals were hypothesized to be partially redundant and were modeled with an interaction term. Temperature and SVPD were modeled with four-knot restricted cubic splines to accommodate possible non-linear relationships with survival time. Separate regression models were fit for *Ae. aegypti* and *Ae. albopictus*. To compare survival times between species, an additional model was fit to the combined data from both species, with a binary indicator variable for species and separate temperature and SVPD splines for each species. Analyses were repeated for all 500 simulation replicates.

Pooled summary models were estimated by drawing ten random normally-distributed values for each model coefficient and simulation replicate using means and standard errors returned by individual stratified model fits; pooled means and confidence intervals were then calculated from the 5,000 accumulated random draws for each coefficient. Summary curves of hazard ratios were constructed in a similar fashion. Results from the Cox regression mixed effects model were summarized using the median and central 95% distribution of the 500 simulation replicates. Heterogeneity among studies was assessed by examining pooled study-level random effects from the mixed effects model.

### Sensitivity analyses

In addition to comparisons between the stratified and mixed effects models, we assessed the robustness of the present study's statistical results via multiple sensitivity analyses. First, results of stratified Cox regression analyses for each study were individually evaluated to assess consistency with the results of the pooled analysis. Next, influences of individual studies on the pooled model were assessed by repeating stratified analyses after excluding each study in turn. As the log-logistic distribution does not meet the proportional hazards assumption underlying Cox regression, the full stratified dual-species analysis was repeated using data simulated only under the Weibull distribution.

## Results

### Study selection and characteristics

**Fig. 2:**
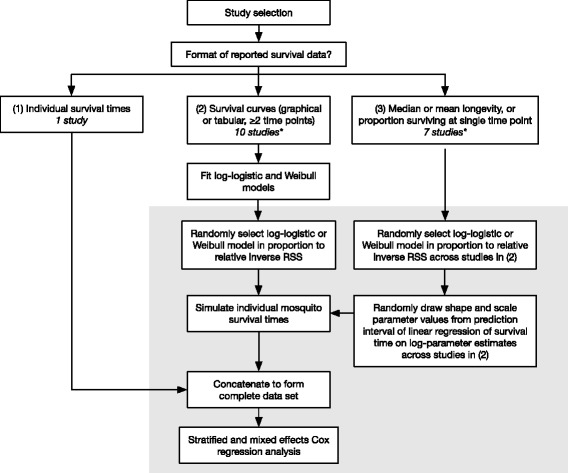
Summary flow diagram of survival time simulations. Steps within the gray box were repeated to generate 500 simulated data sets, which were individually analyzed via stratified and mixed effects Cox regression prior to pooling. (*) One study reported some experiments as survival curves and some as median longevities

A flow diagram for study selection is presented in Additional file 1: Figure S1.1. A total of 1517 unique articles were screened on the basis of both titles and abstracts; 378 articles were assessed for eligibility via full-text review, and 17 studies met eligibility criteria for review. Table 1 summarizes characteristics of the selected studies, and Additional file 2: Table S2 provides further study details. Figure 2 illustrates the data simulation and analytical methodology subsequently used for these studies.

**Table 1:** Summary of studies meeting inclusion criteria for review. Further study details are provided in Additional file 2

Source	Setting	Species^a^	Mosquito origin (Climate zone^b^)	Main exposure variables	Main outcome variables	No. of experiments (total *n*)	Temp. range (°C)	SVPD range (kPa)	Survival data format
Alto et al. [70]	Lab	*Ae. aegypti*, *Ae. albopictus*	Florida, USA (Aw: *Ae. aegypti*; Cfa: *Ae. albopictus*)	Larval density; humidity	Larval survival, development time, adult weight/survival	8 (1150)	23.7–24.1	0.75–1.64	Means, survival curves
Bagny Beilhe et al. [88]	Lab	*Ae. aegypti*	Réunion Island (Af)	Temperature	Multiple life history traits	5 (150)	15–35	0.58–1.41	Medians
Bar-Zeev [66]	Lab	*Ae. aegypti*	na	Temperature; humidity	Egg, larval, pupal and adult survival	28 (1400 minimum)	0.5–40	0.00–7.37	Medians
Beeuwkes et al. [87]	Semi-field	*Ae. aegypti*	Nigeria (Aw)	Site (temperature, humidity); mosquito strain	Adult survival	17 (1495)	22.2–32.8	0.39–2.54	Means
Calado & Navarro-Silva [96]	Lab	*Ae. albopictus*	Brazil (Cfa)	Temperature	Adult survival, fecundity, feeding activity	4 (160)	15–30	0.38–0.95	Means, survival curves
Canyon et al. [92]	Lab	*Ae. aegypti*	Australia (Am)	Nutrition/water; humidity	Adult fecundity, oviposition, survival	7 (350)	27	0.57–2.38	Survival curves (censored)
Canyon et al. [86]	Lab	*Ae. aegypti*	Australia (BSh)	Nutrition; humidity	Adult feeding activity, survival	4 (320)	27.2	0.57–2.38	% mortality at 19 days
Costa et al. [94]	Lab	*Ae. aegypti*	Brazil (Am)	Temperature; humidity	Adult survival, fecundity, fertility	6 (677)	25–35	0.63–2.25	% surviving each day
Delatte et al. [30]	Lab	*Ae. albopictus*	Réunion Island (Af)	Temperature	Multiple life history traits	5 (132)	15–35	0.34–1.12	Plots of fitted Weibull models
Gao et al. [97]	Lab	*Ae. albopictus*	na	Temperature; humidity	Adult survival	8 (2000)	30–35	0.13–5.62	Medians
Goindin et al. [95]	Lab	*Ae. aegypti*	Guadeloupe (Af, Am)	Temperature	Multiple life history traits	3 (363)	24–30	0.60–0.85	Plots/tables of % surviving each day
Hylton [98]	Lab	*Ae. albopictus*	na	Temperature; humidity	Adult survival	14 (*c.*2550)	15.5–32.2	0.18–3.37	Medians, time until 90% mortality
Lewis [91]	Lab	*Ae. aegypti*	na	Temperature; humidity; nutrition	Adult feeding activity, survival	19 (305)	10–30	0.00–2.97	Means
McMeniman et al. [156]	Lab	*Ae. aegypti*	Australia (Aw)	Temperature; density; nutrition	Adult survival	4 (750)	25–30	0.63–0.85	Survival curves
Mogi et al. [89]	Lab	*Ae. aegypti*, *Ae. albopictus*	Indonesia (Af, Am)	Hydration; humidity; mosquito strain	Adult survival	32 (2360)	25	0.32–1.58	Medians, survival curves
Reiskind & Lounibos [90]	Lab	*Ae. aegypti*, *Ae. albopictus*	Florida, USA [Cfa, Af, Am (exact locality uncertain)]	Larval density; humidity	Adult survival	12 (314)	22	0.47–2.05	Means; authors provided raw survival times
Yang et al. [93]	Lab	*Ae. aegypti*	Brazil (Cwa)	Temperature	Multiple demographic parameters	12 (1200)	10–35	0.33–1.58	Means, medians

^a^Some studies examined species in addition to *Ae. aegypti* or *Ae. albopictus*, but these species are not listed

^b^Köppen-Geiger climate classification: Af (tropical-rainforest); Am (tropical-monsoon); Aw (tropical-savannah); BSh (arid-hot steppe); Cfa (temperate-without dry season-hot summer); Cwa (temperate-dry winter-hot summer)

Abbreviation: *na* unspecified, *temp.* temperature

Selected studies collectively reported survival results from 192 unique mosquito cohorts (combining replicates), representing a total of about 15,547 adult female mosquitoes (8749 *Ae. aegypti* and 6798 *Ae. albopictus*). The selected studies as a group were broadly concerned with evaluating relationships between abiotic and biotic factors and mosquito life history parameters (summarized in Additional file 2: Table S2). Study populations had diverse geographical origins and included mosquito strains from temperate, wet tropical and seasonally-dry tropical climates; a single study [86] examined mosquitoes from a hot, arid climate. Only one study [87] examined caged mosquitoes exposed to ambient conditions rather than controlled laboratory conditions. Ten studies reported survival in *Ae. aegypti* alone, four studies focused on *Ae. albopictus*, and three studies reported results for both species. Two studies [88, 89] examined multiple strains of a single species. Most studies reported survival times via graphical survival curves, tables of proportions surviving at multiple time points, single censored observations, or mean or median survival times, though raw survival times were kindly provided by an author of one study [90], and Delatte et al. [30] reported plots of Weibull models fit to observed survival times.

Experimental conditions varied considerably among selected studies. Twelve studies conducted experiments under at least two different relative humidities; seven studies varied both temperature and relative humidity. The distribution of temperature and SVPD values utilized by the reviewed studies is summarized in Additional file 1: Figure S1.2. The range of temperature and humidity conditions used for survival experiments of *Ae. aegypti* were broader than for *Ae. albopictus*: 0.5–40 °C and 0–100% RH (SVPD: 0.00–7.37 kPa) for *Ae. aegypti*, but 15–35 °C and 0–97% RH (SVPD: 0.13–5.62 kPa) for *Ae. albopictus*. Nutrition and hydration provisioning varied widely among studies.

### Qualitative review

Results of individual studies are briefly reviewed here, with further details provided in Additional file 2: Table S2. As some studies varied only temperature or relative humidity across experiments, independent effects on mortality from changes in temperature and SVPD could not be isolated for these studies and discussion may therefore focus only on temperature or humidity.

### *Aedes aegypti*

In an early set of experiments, Lewis [91] found that mean longevity in a stock population of *Ae. aegypti* decreased with increasing temperature (from 10–30 °C) and with increasing SVPD, and was higher in fed than unfed mosquitoes. Beeuwkes et al. [87] compared adult mortality rates in field-collected *Ae. aegypti* from two sites in Nigeria that were exposed to ambient conditions at both sites. The authors detected a site-by-season interaction on mortality (with lowest mortality at temperatures around 27 °C) but suggested that temperature had a greater impact on survival than humidity. Bar-Zeev [66] examined mortality of *Ae. aegypti* under forced starvation and desiccation at a broad range of temperatures and SVPDs and found complex nonlinear relationships among temperature, humidity and mortality. At most temperatures, longevity declined with decreasing humidity, but survival was similar across all humidities at 0.5 °C and 40 °C, suggesting a predominance of thermal effects other than desiccation at extreme temperatures.

Canyon et al. [92] found a reduction in longevity at low (34%) *vs* high (84%) relative humidities in an Australian population of *Ae. aegypti* from a wet tropical climate, while Canyon et al. [86] reported that longevity in an Australian *Ae. aegypti* population from a hot, dry climate depended on the presence or absence of water, sugar or blood sources, with survival similar or greater at low (34%) than high (84%) relative humidities. Yang et al. [93] reported that mortality of adult female *Ae. aegypti* is lowest between about 15–30 °C and increases rapidly at temperatures below or above this range, and Costa et al. [94] reported that longevity in *Ae. aegypti* decreased from 25 °C to 35 °C but was not significantly different at 60 *vs* 80% RH. Goindin et al. [95] found lowest mortality rates for *Ae. aegypti* from Guadeloupe at 27 °C, but only tested a narrow range of temperatures (24–30 °C) and did not systematically vary humidity. Bagny Beilhe et al. [88] reported that developmental rates, survival to adulthood, and adult longevity of *Ae. aegypti* from Réunion were highly temperature-dependent, with greatest adult longevity at 25 °C.

### *Aedes albopictus*

Calado & Navarro-Silva [96] reported that temperature affects adult longevity, fecundity and blood-feeding activity in a temperate-climate *Ae. albopictus* population from Brazil, with different optima for these life history parameters; adult female longevity was highest between 20–25 °C. Gao et al. [97] found that the longevity of adult female *Ae. albopictus* decreases with decreasing humidity (from 97 to 0% RH) at 30 °C and 35 °C. Hylton [98] reported that the longevity of adult female *Ae. albopictus* is affected by temperature and relative humidity, but that these relationships are complex. Within the range of conditions tested, higher temperature was generally associated with reduced longevity, but the relationship with humidity was less clear; at some temperatures, maximum longevity was achieved at intermediate humidities. Delatte et al. [30] found the lowest mortality rates for *Ae. albopictus* from Réunion at 15 °C, with mortality increasing in a complex fashion at higher temperatures up to 35 °C.

### Combined studies

Three studies examined both *Ae. aegypti* and *Ae. albopictus*, enabling direct species comparisons. Mogi et al. [89] reported that *Ae. aegypti* and *Ae. albopictus* strains from Indonesia varied in their desiccation tolerance, that urban strains were more desiccation-tolerant than rural strains, and that *Ae. aegypti* survived longer than *Ae. albopictus*. Alto et al. [70] found significant effects and interactions of species, humidity and larval competition on adult longevity in *Ae. aegypti* and *Ae. albopictus*, with *Ae. aegypti* showing greater longevity than *Ae. albopictus* under most conditions; the direction and scale of the humidity effect on survival was not reported. Reiskind & Lounibos [90] also examined the effect of larval competition on adult survival in both species, and reported greater longevity in *Ae. aegypti* relative to *Ae. albopictus* and reduced longevity in both species with reduced humidity (35 *vs* 85% RH).

### Additional studies

Several additional studies that did not meet the strict criteria for inclusion in our review warrant mention and are described in Additional file 1.

### Survival time simulation, study-level effects and model fit

Among the 73 mosquito cohorts with sufficient reported survival data to fit Weibull or log-logistic models, 16 (22.0%) were best fit by Weibull and 46 (63.0%) by log-logistic models. Eleven cohorts (15.1%) were fit equally well by either model, though these were each represented by observations at only three time points (enabling a perfect fit of either model). Across all 73 cohorts, log-logistic models achieved 59.6% of the weighting used to draw simulation models for the remaining cohorts.

**Fig. 3:**
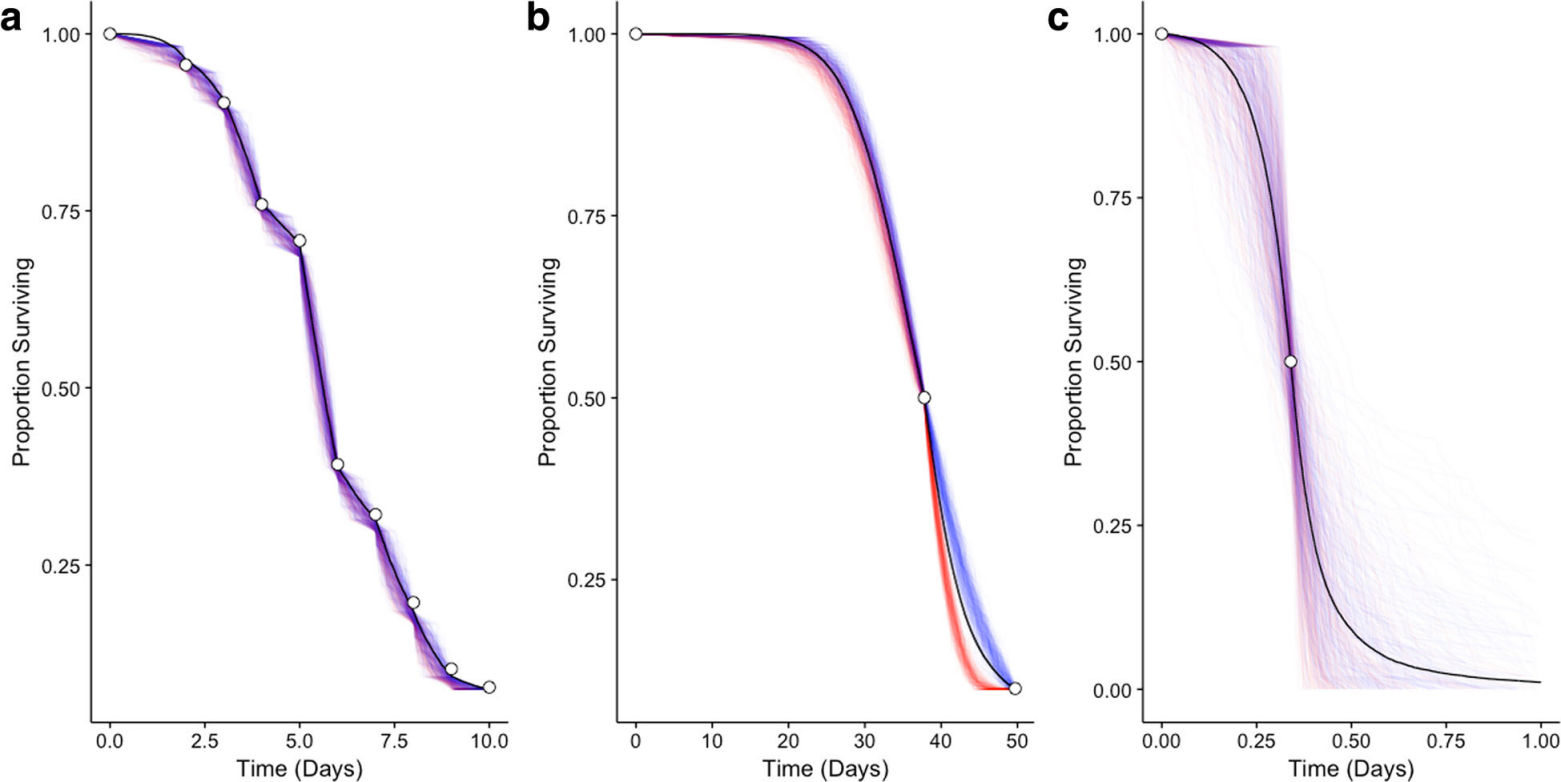
Example simulated survival curves for individual experiments. **a** A right-censored experiment with reported survival observations at numerous time points (*Aedes aegypti* at 24.1 °C and 75% RH, with water but no nutrition supplied; Alto et al. [70]). **b** A right-censored experiment with sparse reported survival observations (*Ae. albopictus* at 32.2 °C and 60% RH, with sugar solution supplied; Hylton [98]). **c** An experiment with only median longevity reported (*Ae. aegypti* at 0.5 °C and 85% RH, with no water or nutrition provided; Bar-Zeev [66]; *x*-axis has been truncated for display purposes). All 500 simulated data sets are shown for each experiment. Survival curves are colored according to their fitted model: Weibull (red), log-logistic (blue), or aggregate (black; all simulated data). Open circles indicate reported observations

Absolute time scales for mosquito mortality varied widely among studies, as illustrated in Fig. 3, which shows survival curves from three experiments as examples of outputs from the simulation process. While much of the variation in longevities is attributable to differences in modeled experimental conditions, the high variance estimated for study-level random effects from the mixed effects Cox regression model (Additional file 1: Figure S1.3) indicates substantial unexplained variability in mortality hazards. Hazard ratios in this context reflect impacts on mortality risk due to study-specific attributes (e.g. experimental procedures or genetic differences between mosquito populations) not captured by other model components. For example, mortality hazards in Bar-Zeev [66] were 12.2 (95% CI: 7.2–20.7) times those predicted by modeled covariates alone, given their average effects across studies. While such large study effects merit further investigation, the use of a stratified Cox regression model for our primary analysis allowed us to accommodate unmodeled study-level differences without assuming a uniform baseline hazard across studies. Although mixed effects models are frequently employed to account for non-independence in pooled analyses, stratification requires fewer statistical assumptions.

Comparisons of relative fits for stratified Cox regression models containing differing sets of covariates, interactions, and functional forms of temperature and humidity are presented in Table 2. Mean differences in AIC values between models across all 500 simulated data sets supported the final model containing non-linear treatment of temperature, non-linear treatment of SVPD, differing temperature and humidity profiles for *Ae. aegypti* and *Ae. albopictus*, and inclusion of sugar, blood and water provisioning. The addition of species-specific non-linear terms for SVPD decreased model AIC values by an average of 2693.11 relative to a model with only temperature and water/nutrition, indicating that humidity significantly improves model fit. Substitution of SVPD with relative humidity or vapor pressure reduced model fit relative to SVPD but provided significant improvements over a model without any humidity term.

**Table 2:** Comparison of Cox regression model fits by mean change over 500 simulated data sets in Akaike Information Criterion (AIC) relative to a reference model of temperature alone (modeled linearly). All models were stratified by study

Model^a^	Mean AIC change
temp	Ref.
temp + blood*sugar + water	-689.97
temp*species + blood*sugar + water	-1328.38
rcs(temp)*species + blood*sugar + water	-2991.04
rcs(temp)*species + SVPD*species + blood*sugar + water	-4693.35
rcs(temp)*species + rcs(SVPD)*species + blood*sugar + water	-5684.15
rcs(temp)*species + rcs(RH)*species + blood*sugar + water	-5371.91
rcs(temp)*species + rcs(VP)*species + blood*sugar + water	-5043.55

^a^temp, temperature (°C); blood, access to blood meals provided; sugar, access to sugar sources provided; water, access to water provided; rcs, restricted cubic spline; SVPD, saturation vapor pressure deficit (kPa); VP, vapor pressure (kPa); RH, relative humidity (%). (*) indicates a modeled interaction. Spline knots were located at the default quantiles in rms (0.05, 0.35, 0.65, and 0.95; Harrell [155]) and correspond to the following values: 10.8, 25.0, 26.2, and 35.0 °C for temperature, and approximately 0.13, 0.63, 1.11, and 3.82 kPa for SVPD (precise knot placements are provided in Additional file 3)

### Temperature, humidity and longevity

**Fig. 4:**
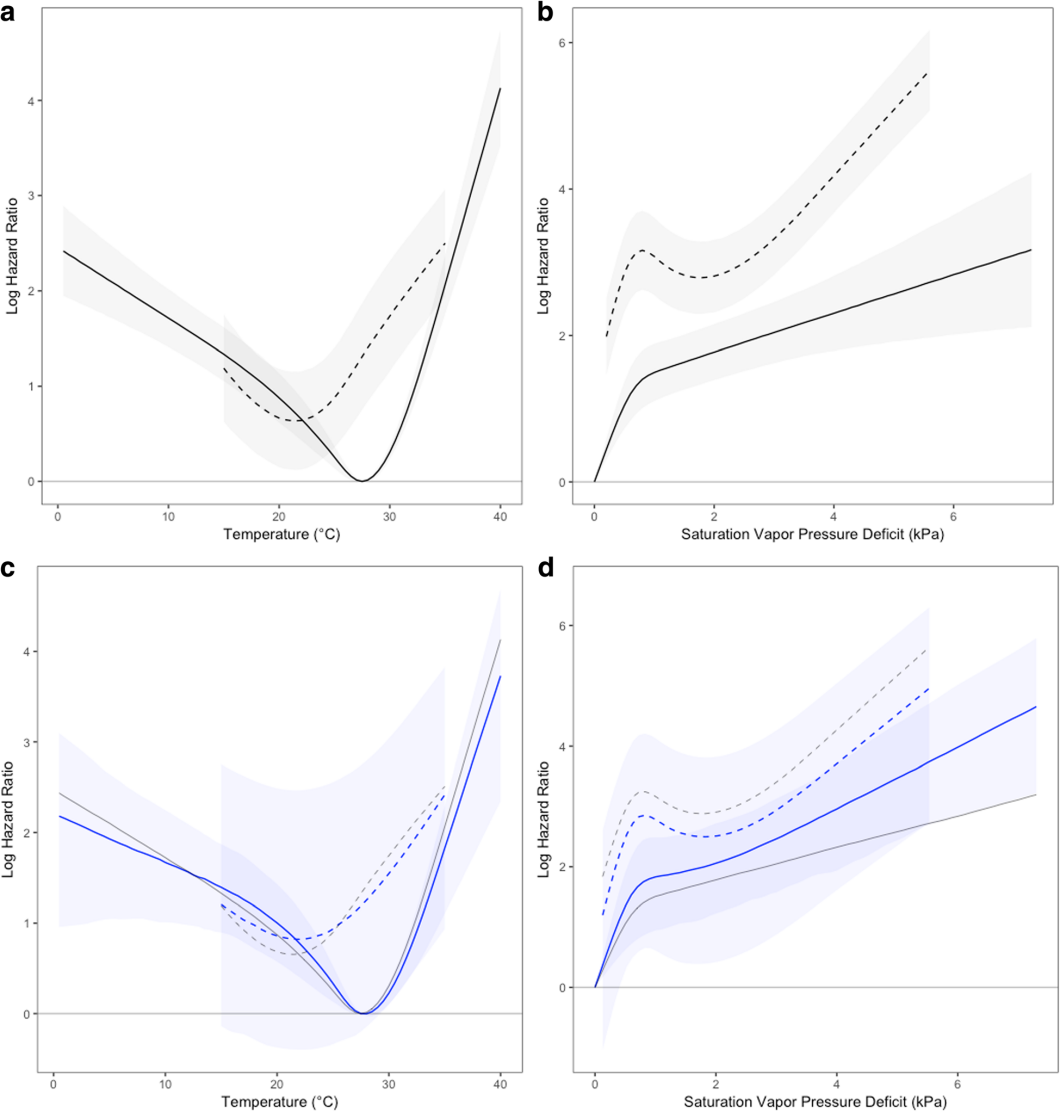
Pooled marginal effects estimates (mean log hazard ratios, with 95% CI) from stratified model analysis for temperature (**a**) and saturation vapor pressure deficit (**b**), and from mixed effects model analysis for temperature (**c**) and saturation vapor pressure deficit (**d**). Results for *Aedes aegypti* are indicated with solid lines, and for *Ae. albopictus* by dashed lines. Estimates in **a** and **c** are relative to a reference of *Ae. aegypti* at 27.5 °C, and in **b** and **d** are relative to a reference of *Ae. aegypti* at full saturation (100% RH). Plots in **c** and **d** provide median (blue lines) and 95% CI (blue shading) from the mixed effects model, with mean estimates from stratified analyses provided for comparison (gray lines). Estimates are restricted to the range of values present in the data for each species

As estimated species-specific hazard curves for temperature and SVPD were highly consistent between single- and dual-species Cox regression analyses, reporting of results focuses on the dual-species model as it allows direct comparison between *Ae. aegypti* and *Ae. albopictus*. The full specification of the pooled stratified model is presented in Additional file 3. Estimated temperature-related mortality profiles from the stratified model differed between *Ae. aegypti* and *Ae. albopictus* (Fig. 4a), with *Ae. aegypti* estimated to have a species-specific temperature optimum (i.e. lowest mortality hazard) at a higher temperature than *Ae. albopictus* (27.5 °C *vs* 21.5 °C, respectively). Results suggest that *Ae. albopictus* may have a small survival advantage relative to *Ae. aegypti* between 15 °C (the lower bound of the available data for *Ae. albopictus*) and about 22 °C, though the differences are not statistically significant. *Aedes aegypti* was estimated to have a significant survival advantage over *Ae. albopictus* above 22 °C, though possible convergence is evident around 35 °C. For both species, mortality risk increases gradually as temperatures decrease from the species optima but rise rapidly with increasing temperature. Median estimates of temperature-related mortality hazards from the mixed effects model (Fig. 4c) were similar to the stratified model, but with substantially higher uncertainty (especially for *Ae. albopictus*), such that differences between species were not significant in this analysis.

Mortality profiles from the stratified model also differed between species for SVPD (Fig. 4b), with *Ae. albopictus* showing higher mortality than *Ae. aegypti* at all SVPD values modeled for *Ae. albopictus* (as with temperature, these estimates include the main effects for species). For both species, hazard ratios (HRs) increase rapidly from 0.0 kPa to around 1.0 kPa, then increase more gradually as SVPD increases. The log HR curve for *Ae. albopictus* displays a marked temporary decrease between roughly 1.0 kPa and 2.0 kPa, which may suggest overparameterization of the spline function or could reflect a true aspect of the vapor pressure-survival relationship for this species. Relative to the stratified model, the mixed effects model estimated a more rapid rise in mortality hazard for *Ae. aegypti* with increasing SVPD (Fig. 4d), less pronounced median differences between *Ae. aegypti* and *Ae. albopictus*, and much wider confidence intervals for both species. Differences in SVPD-mortality profiles were not significant between species in the mixed effects model.

**Fig. 5:**
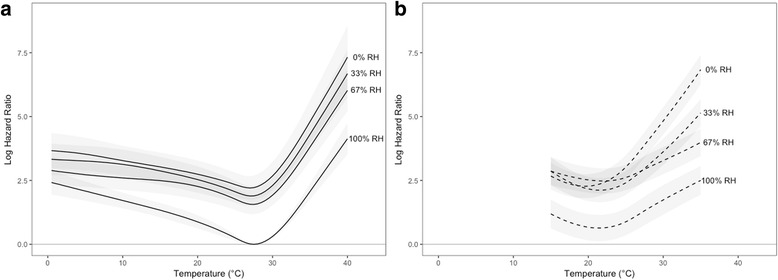
Pooled joint effects estimates (mean log hazard ratios, with 95% CI) from stratified model analysis for temperature and select relative humidities (%) for *Aedes aegypti* (**a**) and *Ae. albopictus* (**b**), illustrating the interacting effects of temperature and humidity. In order to facilitate direct comparisons between species, estimates for both species are relative to a reference of *Ae. aegypti* at 27.5 °C and full saturation (100% RH)

The combined effects of temperature and humidity on adult mortality are illustrated for the stratified model in Fig. 5 for RH terciles. For both species, the largest relative increase in mortality hazard occurs between 100% RH (saturation) and 67% RH at most temperatures. For example, hazard ratio estimates for *Ae. aegypti* at 27.5 °C are 4.76 (95% CI: 3.26–6.97) at 67% RH, 6.65 (95% CI: 4.48–9.86) at 33% RH and 9.18 (95% CI: 5.34–14.70) at 0% RH, relative to saturation. The estimated influence of humidity (desiccation) on mortality hazards increases steadily for *Ae. aegypti* with increasing temperature (Additional file 1: Figure S1.4), and similarly increases for *Ae. albopictus* above about 25 °C. The main effect term for species revealed significantly higher mortality in *Ae. albopictus* relative to *Ae. aegypti* in the stratified model (HR: 4.01; 95% CI: 1.15–14.01) but not in the mixed effects model (HR: 1.87; 95% CI: 0.89–3.93). Pooled estimates for other covariates in the model revealed significant survival benefits from provisioning of water (stratified model: HR: 0.17; 95% CI: 0.11–0.24; mixed effects model: HR: 0.20; 95% CI: 0.18–0.23), sugar (stratified model: HR: 0.04; 95% CI: 0.02–0.24; mixed effects model: HR: 0.06; 95% CI: 0.04–0.11) and blood meals (stratified model: HR: 0.12; 95% CI: 0.08–0.19; mixed effects model: HR: 0.18; 95% CI: 0.12–0.25), but the simultaneous provisioning of sugar and blood did not improve survival relative to sugar alone (stratified model: HR for sugar-blood interaction: 8.94 [essentially the inverse of HR for blood meals]; 95% CI: 5.47–14.59; mixed effects model: HR: 6.09; 95% CI: 4.08–9.10; this result contrasts with that of Styer et al. [99]).

### Sensitivity analyses

Stratified Cox regression analyses of simulated survival times for most individual studies were in approximate agreement with the pooled results (Additional file 4: Table S4.1, Figures S4.1-S4.4), but some studies yielded hazard curves that were inconsistent with the pooled analysis. For example, the *Ae. aegypti* temperature curve derived from Lewis [91] suggests a monotonically and linearly increasing mortality hazard between 10–30 °C, and data from both Canyon et al. [86] and Reiskind & Lounibos [90] suggest decreasing mortality for *Ae. aegypti* with increasing SVPD. Data from a few studies [30, 93, 95] imply non-linear SVPD-mortality curves within narrow SVPD ranges. Results from Bar-Zeev [66] most closely matched the pooled SVPD hazard curve for *Ae. aegypti*, and the pooled model was generally robust to exclusion of this study except at temperature and SVPD extremes. Only Bar-Zeev’s experiments included data at these extremes and model estimates in these ranges were reliant on these observations (Additional file 4: Figures S4.5-S4.6). It is notable that, although the results of the only semi-field study included in this review (Beeuwkes et al. [87]) are potentially influenced by highly variable field conditions and by inconsistent nutritional provisioning among experiments, results of that study are highly compatible with the pooled model, and the latter is robust to exclusion of the Beeuwkes data. Exclusion of other individual studies had minimal impact on the results of pooled analyses. Stratified analysis of survival data simulated entirely under the Weibull model yielded results similar to those from the combined Weibull/log-logistic models (Additional file 4: Figure S4.7).

A completed PRISMA checklist is provided in Additional file 5.

## Discussion

To our knowledge, this study represents the first systematic review and pooled analysis of simultaneous associations of temperature and humidity with survival in adult female *Ae. aegypti* and *Ae. albopictus*. We identified 17 studies that reported experimental results with sufficient methodological detail and experimental scope to enable qualitative and quantitative assessments of these relationships. Reviewed studies collectively supported a temperature-dependent relationship between humidity and adult female survival in *Ae. aegypti* and possibly *Ae. albopictus*, with the effect modified by factors including nutrition and hydration provisioning, larval competition, and probably genetics (i.e. population-level differences). Stratified survival analysis estimated the lowest relative mortality hazards for *Ae. aegypti* and *Ae. albopictus* around 27.5 °C and 21.5 °C, respectively, with pronounced increases in mortality risk at lower and (especially) higher temperatures. Mortality was estimated to increase non-linearly with increasing desiccation stress in both species, corresponding with greater effects of aridity on mortality at higher temperatures. The incorporation of data from three studies [70, 89, 90] that assessed survival for both *Ae. aegypti* and *Ae. albopictus* allowed direct estimation of relative species-specific mortality risks in our study, with other experimental factors controlled (see Additional file 4: Figure S4.8 for further discussion). Estimated adult mortality risks in the stratified analysis were higher for *Ae. albopictus* than for *Ae. aegypti* at all modeled vapor pressure deficits and at most modeled temperatures, though mortality hazards did not differ significantly between species in the mixed effects model. As relative measures, our model results can be directly used to modify survival functions in dynamic models, or to predict longevity in laboratory or field-based populations for which baseline survival trends have been measured.

The critical roles of temperature in *Aedes* population dynamics and associated disease transmission have been widely reported and frequently incorporated into statistical and mathematical models (e.g. [10, 11, 64, 65, 100]). Recently, Brady et al. [34] used generalized additive models (GAMs) to estimate the temperature-mortality relationship for adult *Ae. aegypti* and *Ae. albopictus* from field- and laboratory-based experiments. Their models estimated optimal temperatures for *Ae. aegypti* around 21 °C and for *Ae. albopictus* around 24.5 °C under controlled laboratory conditions, with an overall survival advantage for *Ae. albopictus* except at extreme high and low temperatures within the modeled 0–40 °C range. These results contrast with those from our stratified model, in which *Ae. aegypti* had its lowest mortality around 27.5 °C, *Ae. albopictus* had optimum survival around 21.5 °C, and *Ae. aegypti* had lower mortality risk than *Ae. albopictus* at most temperatures (although our model does not estimate mortality for *Ae. albopictus* across the full temperature range). The contradictory results between our study and those of Brady et al. [34] may reflect our inclusion of humidity as a modifier of the temperature-survival relationship, or differences in study selection and analytical approach. Model estimates of a lower temperature optimum for *Ae. albopictus* are consistent with the ecological niche models (e.g. [101, 102]) suggesting that the geographical range of *Ae. albopictus* extends to cooler climates than that of *Ae. aegypti*. For example, Johnson et al. [102] used a MaxEnt model to relate USA county-level *Ae. aegypti* and *Ae. albopictus* presence to climatic variables independently of laboratory data, and estimated that the range of *Ae. albopictus* extends to cooler areas than *Ae. aegypti* can tolerate.

Both *Ae. aegypti* and *Ae. albopictus* are highly invasive vector species, and previous research has documented competitive interactions between these species at the larval stage, local replacement of *Ae. aegypti* by *Ae. albopictus*, and differences in their ecological niches (e.g. [101, 103–107]). These dynamics underscore the complexity of the environmental-mosquito interactions that influence species establishment and survival. The high uncertainty in mortality estimates for *Ae. albopictus* and the absence of statistically significant mortality differences between species in our mixed effects model caution against over-interpretation of species differences in our stratified model. However, consideration of the relationship between humidity and adult survival in both *Ae. aegypti* and *Ae. albopictus* may provide future insights into the invasion and population ecology of both species, for example by refining ecological niche models and improving understanding of the role of urbanicity in enabling *Aedes* persistence in otherwise marginal environments (e.g. [101, 108, 109]). While our study specifically addresses the impact of temperature and humidity on adult longevity, relative mortality rates in these species are likely to be highly context-dependent, and the ability of either species to establish and thrive in a particular habitat further depends on other processes affecting adults as well as egg, larval and pupal stages [10, 20, 30, 31, 33, 40, 67, 70, 91, 92, 96, 104, 110, 111]. Notably, laboratory experiments indicate that *Ae. albopictus* can outcompete *Ae. aegypti* when cohabitating in the immature stages (e.g. [112, 113]).

Compared with temperature, relatively little attention had previously been devoted to modeling the effect of humidity on adult survival in *Aedes* species. Focks et al. [10, 11], and derivative dynamic mosquito population models including Skeeter Buster [14, 65, 114, 115], modeled the relationship between adult survival and SVPD for *Ae. aegypti* using a simple function that linearly reduces daily adult survival rates by 40% between 1.0 and 3.0 kPa SVPD, with stable mortality rates below and above this range. Our results contrast with this model in estimating significant mortality increases between 0.0 and 1.0 kPa and above 3.0 kPa, perhaps reflecting our study’s flexible modeling strategy and inclusion of additional data sources. Surprisingly, our model estimated the most rapid increase in mortality risk for *Ae. aegypti* between 0.0 and roughly 1.0 kPa SVPD, with a generally slower increase in mortality above 1.0 kPa SVPD. This unexpected result may reflect greater impacts on mortality from thermal processes other than desiccation at high temperatures. Lega et al. [64] also incorporated a simple humidity-dependent adult mortality function for *Ae. aegypti* in the Dynamic Mosquito Simulation Model (DyMSiM; [15]). In their model, daily probability of adult survival is increased from 0.91 to 0.98 when RH is between 72% and 95% at temperatures from 4 °C to 41 °C. The results of Lega et al. [64] demonstrated that the addition of a simple humidity-survival term can improve model fit to field data relative to temperature alone.

Mathematical *Aedes* models have frequently relied on temperature or humidity thresholds to set adult mortality rates, often at fixed levels or according to a linear step function (e.g. [10, 20]). Our results provide an opportunity to further refine these models by flexibly accounting for the non-linear and temperature-dependent relationship of humidity with adult survival. Because our equations are relative (i.e. expressed as hazard ratios), they have the flexibility to either augment or replace existing equations in dynamical models. Properly representing humidity effects in dynamical *Aedes* models is an increasingly important issue to address in a changing climate, as observed water vapor levels in the atmosphere have increased in recent decades [116] and are projected to keep increasing throughout this century as temperatures continue rising globally [117]. Yet, because of the relationship of humidity with temperature, the manner in which humidity is quantified is important. To explain, while overall vapor pressure (VP) generally increases under warming, relative humidity (RH) generally remains about constant, and saturation vapor pressure deficit (SVPD) generally increases (i.e. desiccation potential rises) [118, 119]. Therefore, an analysis of the impacts of humidity on *Aedes* survival under changing climatic conditions could potentially conclude that desiccation stress decreases, remains constant, or increases, depending on whether VP, RH, or SVPD is used as the humidity variable. In our analysis, SVPD provided a better model fit to survival data than either RH or VP. On its own, RH may be a poor metric of humidity for epidemiological models of infectious disease because it reflects an absolute measure of humidity only within the context of a given temperature [120]. By contrast, SVPD is a good proxy for desiccation stress because it correlates with evaporation rates and therefore measures the drying capacity of air [121]. For example, 25% RH at 5 °C (SVPD: 0.65 kPa) represents a more similar desiccation environment to 75% RH at 5 °C (SVPD: 0.22 kPa) than to 25% RH at 40 °C (SVPD: 5.53 kPa).

While the sole use of experimental data in our study strengthens inferences of a causal effect of humidity on mortality, caution is warranted in making such interpretations given the presence of residual unexplained variation in mortality rates, as reflected by the large study-level effects estimated by our mixed effects model. This variation is likely due to unmeasured or unreported factors affecting mosquito lifespan, including experimental conditions and/or species variants that we could not include in the model. In the laboratory setting these factors likely include genetics, diurnal temperature and humidity ranges, mating and oviposition activity, light-dark cycles, nutritional quality and frequency, intra- and interspecific larval competition, and adult density. Specific factors that could introduce uncertainty into the estimated humidity-longevity relationship include high variability of temperature and/or humidity within single experiments; inconsistent timing or quality of nutrition or hydration provisioning among experiments; provision of nutrition prior to the start of experiments; use of mosquitoes greater than one day post-eclosion for experiments; prevention of mating and oviposition; and use of stock colonies that had been maintained in captivity for many generations. These factors could not be reliably included in the statistical analyses due to inconsistent reporting across studies (summarized in Additional file 4: Table S4.1), but additional experimental and analytical attention would likely improve the precision and reliability of both relative and absolute survival estimates.

Our analysis was limited by the difficulty in modeling the large variation among studies in timing, quality and frequency of water and nutritional provisioning. We modeled these factors as simple binary presence/absence variables, and hence provide only a simple contrast between complete desiccation or starvation and availability of any hydration or nutrition sources post-eclosion. However, blood meal source and quality have been shown to influence *Aedes* adult longevity [122, 123]; larval nutrition also impacts adult longevity in *Ae. aegypti* and *Ae. albopictus* [70, 124] and influences their desiccation resistance [125]. Despite its demonstrated influence on adult longevity in laboratory experiments (e.g. [86, 126]), sucrose-feeding may be a relatively insignificant source of nutrition for adult female *Ae. aegypti* in the field (e.g. [127]), and its impact on desiccation tolerance in field populations requires further study. More nuanced analyses of *Aedes* adult survival in relation to these and other nutrition-related variables may further improve population and disease modeling efforts.

The diverse geographical origins of *Aedes* populations in the reviewed studies imply significant collective genotypic and phylogenetic diversity among study populations, though the degree to which they represent the global diversity of *Ae. aegypti* and *Ae. albopictus* is unknown. Some studies performed experiments using stock colonies that had been in captivity for many generations, increasing the likelihood of adaptation to laboratory conditions in these strains and potentially reducing their desiccation tolerance relative to field populations. A majority of strains with reported collection localities hailed from regions with tropical climates, and only a single study used mosquitoes collected from a hot, arid locality [86]. Interestingly, the results from this latter study in arid Australia contrasted with results from the same author using a tropical population, with those from the tropical environment showing a greater impact of lower humidity on longevity, while those from an arid environment were robust when provisioned. Humidity is likely to be an important driver of mosquito dynamics in seasonally dry tropical environments, such as those with monsoon climates [76], to which *Ae. albopictus* has been hypothesized to have adapted prior to its global spread [128]. The poor representation of *Aedes* populations from arid and semi-arid regions is an important limitation of the present study. Studies assessing variation in desiccation tolerance among *Aedes* populations are scarce, and differences among strains used by studies in our review could not be completely isolated from potential confounders. Most laboratory studies investigating multiple conspecific populations of *Ae. aegypti* or *Ae. albopictus* have found meaningful population-level variation in mortality rates [87, 89, 110, 129], though Machado-Allison & Craig [130] only found significant differences between sylvan and urban lineages. Ecological and epidemiological *Aedes* models should aim to account for the unique genotypic and phenotypic profiles of mosquito populations of interest, which can display significant genetic isolation and differentiation even over relatively short distances [131–133]. Our analysis provides a baseline from which humidity effects on adult survival can be better incorporated into *Aedes* models, but careful studies of heat and desiccation tolerance in drylands *Aedes* populations would likely improve model accuracy in such regions.

*Aedes aegypti* distribution is variable across time and space and has been associated with small-scale habitat, climate and human demographic factors (e.g. [35, 104, 134, 135]). The existence of significant microclimatic variability in urban environments [58, 136–138], possible behavioral responses by mosquitoes to suboptimal conditions [86, 92, 139], and the modulating effect of humidity on adult *Aedes* behavior (e.g. [140]) further suggest that macroclimatic measurements may be unreliable proxies for conditions experienced by individual mosquitoes in the field. Even when ambient temperature and humidity are not conducive for survival, it may be possible for *Aedes* females to exploit gentler microhabitats. We hypothesize that compensatory behavioral responses to temperature and desiccation stress could loosen the tight linkage between environmental conditions and *Aedes* mortality estimated by laboratory experiments. Under this hypothesis, desiccation and temperature stresses may be most appropriately viewed as constraints on adult *Aedes* females, which could manifest as changes in survival, feeding, reproduction, or dispersal activities, depending on circumstances. Incorporation of the humidity-survival relationships estimated by the present study into dynamic mosquito models would represent an important step toward improving forecasts of *Aedes*-associated disease transmission in arid, semi-arid and seasonally dry regions. Further insights could emerge from modeling strategies that account for genotypic and phenotypic variation and evolution within *Aedes* populations (e.g. [141]), ecological heterogeneity and the effects of fluctuating environmental conditions, and local processes affecting mosquito physiology and behavior. Agent-based models are one such promising avenue of research [142–145].

Most studies included in the present review were performed in controlled laboratory environments. Relatively few studies have rigorously examined changing field conditions and adult longevity, making it difficult to assess how the findings from the laboratory studies might apply to natural mosquito populations. Short-term temperature fluctuations have important effects on life history traits and viral transmission in *Ae. aegypti* [111, 146–148] (reviewed for insects generally by Colinet et al. [149]). For example, laboratory experiments with *Ae. aegypti* have demonstrated reduced immature development rates, adult female longevity, fecundity, and rates of infection by DENV at higher diurnal temperature ranges (DTRs) [111, 146]. The effects of fluctuating humidity may also have important effects on mortality, however these have not been extensively investigated. Lansdowne & Hacker [110] examined the effect of naturally varying temperature and humidity regimes on adult *Ae. aegypti* survival and did not find significant differences relative to constant conditions, though the contribution of humidity in their study cannot be separated from thermal effects. Models of *Aedes* survival may benefit in the future from careful laboratory studies of *Aedes* mortality under fluctuating humidities and constant temperatures. In modeling the relationship between temperature and survival in *Ae. aegypti* and *Ae. albopictus*, Brady et al. [34] included survival data from mark-release-recapture studies in order to quantify the substantial differences in mortality between laboratory and field populations. However, our review failed to identify fully field-based studies with sufficient humidity data for reliable inclusion in our model.

Differences in reported survival data formats presented a challenge to identifying uniform summary survival measures across studies. The wide range and varying overlap of experimental conditions among studies, and the hypothesized nonlinear relationships of temperature and humidity with longevity, further precluded the use of standard meta-analysis tools. Our approach enabled an integrated analysis of these disparate survival data, and accommodated non-linear effects of temperature and humidity, measured and unmeasured heterogeneity in study design, and age-dependent mortality, which has been detected for *Aedes* in both field and laboratory studies (e.g. [34, 149–152]). Survival time simulations represent a potential source of bias for experiments in which only a mean or median survival time was reported, as the shapes of their simulated survival curves may be biased toward those found in studies with fitted models. However, by simulating 500 data sets per experiment, with parameters drawn from the wide prediction intervals of the fitted models, we effectively increased model variance for low-reliability experiments and assigned greater weight to more reliable studies. Furthermore, as our stratified modeling strategy was designed to emphasize estimation of effects within studies, simulation of mosquito longevities is only likely to introduce significant bias if baseline hazards differ substantially among experiments within an individual study, for example when conditions are inconsistently variable.

Our simulations assumed Weibull or log-logistic distributions for survival times (other distributions are also reasonable, e.g. [34, 152–154]). Styer et al. [99] found that the logistic distribution provided the best fit for their large survival cohort of *Ae. aegypti*, though they did not evaluate the Weibull or log-logistic distributions. Brady et al. [34] reported that no single distribution best fit their survival data for *Ae. aegypti* and *Ae. albopictus* in all circumstances, but that the log-logistic and exponential distributions generally provided the best fits for *Ae. aegypti* and *Ae. albopictus*, respectively. We used the Weibull model (of which the exponential distribution is a special case) for its proportional hazards properties, and the log-logistic model because it provided comparable or better fit to our data. Bias is likely to be a concern only if true survival curves varied significantly in form among cohorts within a given study. Even in such cases, estimated hazard ratios can productively be interpreted as average effects over time, with non-proportionality indicating time-varying effects of covariates on mortality rates, or additional unmodeled factors influencing survival. These concerns can best be addressed through future studies examining raw (non-simulated) survival times in Cox regression or accelerated failure time models [155].

The analytical results of this study were widely robust to exclusion of individual studies, and single-study analyses were generally, though not universally, consistent with the pooled results. Given the variable presence of unmodeled experimental factors or interactions that may modify the associations of temperature and humidity with survival, we should not expect a priori that all individual studies will conform precisely to the results of a simple pooled model. For example, Canyon et al. [86] found that *Ae. aegypti* females provided access only to water did not imbibe water when maintained at 84% RH (27.2 °C) but did so at 34% RH, yielding faster mortality at the higher humidity; this result suggests the existence of an important interaction that was not included in our model and partially explains the discrepancy of the Canyon et al. [86] and pooled SVPD models. Some inconsistencies with the pooled model could result from the simpler forms of the single-study models, for example in studies where temperature and SVPD could not be modeled simultaneously or where too few conditions were examined to enable non-linear modeling. Other discrepancies may result from the finely-resolved spline models for some individual studies, which yield more complex SVPD-mortality curves than the pooled model, which was designed to yield a smooth hazard curve across a broad range of conditions. The causes of significant deviations from the pooled model warrant future investigation to identify other important factors affecting the relationships among temperature, humidity and survival. Despite the lack of universal concordance, a large proportion of single-study models were in basic agreement with the pooled model. In addition, the single-study hazard curves highlight the limited ranges of temperature and humidity examined by most studies, and demonstrate the utility of our analytical approach in combining overlapping curves to derive hazard estimates over a broader range. Statistical confidence in the stratified model was lowest at extreme temperatures and at high SVPD, reflecting a relative scarcity of experimental data and low reliability of simulations for most studies conducted under these conditions. It is vital that additional studies are conducted to fill these gaps given the growing importance of *Aedes*-borne viruses in arid environments. It is worth noting that, although the majority of evaluated studies did not meet our strict inclusion criteria, a great many studies reported experimental data that could inform aspects of *Aedes* survival beyond those on which we focused, or within other contexts or analytical frameworks.

Assessment of publication bias was not straightforward, but studies included in the review addressed a wide range of research questions, with just over half explicitly examining the association of humidity with survival. The high frequency of other primary research questions suggests a lower risk of publication bias in this body of work. A wide variety of search engines were used and no language or time restrictions were made on the search. While most of the databases we searched are biased toward publications in English, the inclusion of studies written in Mandarin and Portuguese (one study each) indicates that the search did not solely capture English-language publications. In addition, the geographical coverage of lead institutions indicates a fairly wide distribution of countries in the analysis.

Our analyses were limited by the availability of published literature reporting the associations among longevity, humidity and temperature. We identified gaps in the published literature that should be filled to improve our understanding of these dynamics. Few studies have examined *Aedes* mortality under arid conditions or at extreme temperatures, and relatively few studies have carefully examined the effect of humidity on *Ae. albopictus* mortality. Difficulties in accounting for microclimatic variations in temperature and humidity in field studies complicate the reliable estimation of relative humidity-associated mortality hazards in field *vs* laboratory populations. Finally, experimental data for genetic variation in desiccation tolerance in *Ae. aegypti* and *Ae. albopictus* are scarce, preventing a detailed understanding of the evolutionary potential for adaptation to aridity within populations in marginal environments or in response to climatic change.

## Conclusions

Our systematic review and pooled survival analysis revealed strong evidence for temperature-dependent and non-linear associations of humidity with adult female survival in *Ae. aegypti* and possibly *Ae. albopictus*, important vectors of major human pathogens including dengue, chikungunya, yellow fever and Zika viruses. *Aedes aegypti* was found to have greater longevity than *Ae. albopictus* at most temperatures and humidities and to have a higher optimum temperature, consistent with the current geographical distributions of these species. Our quantitative models may facilitate improved vector and disease forecasts across a range of spatial and temporal scales in arid, semi-arid, and seasonally dry environments. More robust modeling of mosquito responses to desiccation and temperature stress could also support projection of impacts from climate change and urbanization on the risks of *Aedes*-borne viral transmission.

## Additional files

Additional file 1: **Text 1.** List of search strings used in each database for literature search. **Figure S1.1.** Flow diagram for study selection. Many studies were excluded based on multiple criteria but are tabulated here under only one criterion by which they were deemed ineligible. **Figure S1.2.** Distribution of mean temperature and saturation vapor pressure deficits employed by studies included in the present review. Circles represent individual experiments and are colored by species: *Aedes aegypti* (blue, without borders) or *Ae. albopictus* (green, with gray borders). Circle size is proportional to total sample size for an experiment. Lines represent 0% (upper line) and 100% (lower line) RH. **Figure S1.3.** Study-level random effects from mixed effects Cox regression model for studies included in the present review. Estimates and 95% confidence intervals are pooled across all 500 simulated data sets and reflect study-specific differences in mortality hazards after conditioning on modelled covariates. **Figure S1.4.** Pooled joint effects estimates (mean log hazard ratios, with 95% CI) from stratified model analysis for select relative humidities (%), relative to saturation (100% RH) at a given temperature, for (**a**) *Aedes aegypti* and (**b**) *Ae. albopictus*. Results reflect the temperature- and species-dependent associations of desiccation with mortality risk. **Table S1.1.** Ascertainment of potential sources of error or uncertainty in included studies. **Text 2.** Additional studies of note. (DOCX 453 kb)

Additional file 2: **Table S2.** Summary of the methods, results and conclusions of the 17 studies included in the present review. (format: .xlsx) (XLSX 60 kb)

Additional file 3: Model specification of the full dual-species Cox regression model, with pooled coefficient estimates. (PDF 103 kb)

Additional file 4: **Table S4.1.** Single-study models used for sensitivity analyses assessing consistency of pooled model results with individual studies. Models represent the most complex treatment of variables in the pooled model possible for a given study. **Figure S4.1**. Comparison of model estimates for pooled (black) and single-study (blue) analyses of log hazard ratios (with 95% CI) by temperature (°C) for *Aedes aegypti*. Pooled model estimates are relative to a reference of *Ae. aegypti* at 27.5 °C. **Figure S4.2.** Comparison of model estimates for pooled (black) and single-study (blue) analyses of log hazard ratios (with 95% CI) by temperature (°C) for *Aedes albopictus*. **Figure S4.3.** Comparison of model estimates for pooled (black) and single-study (blue) analyses of log hazard ratios (with 95% CI) by saturation vapor pressure deficit (kPa) for *Aedes aegypti*. Pooled model estimates are relative to a reference of *Ae. aegypti* at full saturation. **Figure S4.4.** Comparison of model estimates for pooled (black) and single-study (blue) analyses of log hazard ratios (with 95% CI) by saturation vapor pressure deficit (kPa) for *Aedes albopictus*. **Figure S4.5.** Comparison of model estimates from all studies (black) and with individual studies excluded (blue) of log hazard ratios (with 95% CI) by temperature (°C) for *Aedes aegypti* (solid lines) and *Ae. albopictus* (dashed lines). Model estimates are relative to a reference of *Ae. aegypti* at 27.5 °C. **Figure S4.6.** Comparison of model estimates from all studies (black) and with individual studies excluded (blue) of log hazard ratios (with 95% CI) by saturation vapor pressure deficit (kPa) for *Aedes aegypti* (solid lines) and *Ae. albopictus* (dashed lines). **Figure S4.7.** Comparison of model estimates after simulation under mixed Weibull and log-logistic survival time distributions (black, bold lines) and under the Weibull distribution alone (blue, thin lines) for (**a**) temperature and (**b**) saturation vapor pressure deficit. Estimates are shown for *Aedes aegypti* (solid lines) and *Ae. albopictus* (dashed lines), and are relative to a reference of *Ae. aegypti* at 27.5 °C (A) or *Ae. aegypti* at full saturation (B). **Text 1.** Relative mortality hazards between *Ae. aegypti* and *Ae. albopictus*. (DOCX 1290 kb)

Additional file 5: Completed PRISMA checklist. (DOC 64 kb)

## Abbreviations

AIC: Akaike’s information criterion
CHIKV: Chikungunya virus
DENV: Dengue virus
DTR: Diurnal temperature range
EIP: Extrinsic incubation period
kPa: Kilopascal
RH: Relative humidity
RSS: Residual sum of squares
SVPD: Saturation vapor pressure deficit
VP: Vapor pressure
YFV: Yellow fever virus
ZIKV: Zika virus
