# Supplementary material for: Effects of desiccation stress on adult female longevity in Aedes aegypti and Ae. albopictus (Diptera: Culicidae): results of a systematic review and pooled survival analysis
Source: Parasit Vectors. 2018 Apr 25;11:267. doi: 10.1186/s13071-018-2808-6 (PMC5918765; doi:10.1186/s13071-018-2808-6)
Supplement: Supplementary file 1 — Text 1. List of search strings used in each database for literature search. Figure S1.1. Flow diagram for study selection. Many studies were excluded based on multiple criteria but are tabulated here under only one criterion by which they were deemed ineligible. Figure S1.2. Distribution of mean temperature and saturation vapor pressure deficits employed by studies included in the present review. Circles represent individual experiments and are colored by species: Aedes aegypti (blue, without borders) or Ae. albopictus (green, with gray borders). Circle size is proportional to total sample size for an experiment. Lines represent 0% (upper line) and 100% (lower line) RH. Figure S1.3. Study-level random effects from mixed effects Cox regression model for studies included in the present review. Estimates and 95% confidence intervals are pooled across all 500 simulated data sets and reflect study-specific differences in mortality hazards after conditioning on modelled covariates. Figure S1.4. Pooled joint effects estimates (mean log hazard ratios, with 95% CI) from stratified model analysis for select relative humidities (%), relative to saturation (100% RH) at a given temperature, for (a) Aedes aegypti and (b) Ae. albopictus. Results reflect the temperature- and species-dependent associations of desiccation with mortality risk. Table S1.1. Ascertainment of potential sources of error or uncertainty in included studies. Text 2. Additional studies of note. (DOCX 453 kb) [file 13071_2018_2808_MOESM1_ESM.docx]

**Additional file 1**

**Text 1. Literature Search Strategy**

Web of Science (ALL DATABASES): "TS=((Aedes OR Stegomyia OR Aedinae) AND (humid* OR arid* OR 'vapor pressure' OR desiccat* OR precipitation OR temperature OR 'water stress' OR climat* OR weather) AND (surviv* OR longevity OR lifespan OR 'life span' OR 'life expectancy' OR mortality))" [893 hits] [7 February, 2016]

PubMed: "(((aedes OR stegomyia OR aedine) AND (humid* OR arid* OR vapor pressure OR desiccat* OR precipitation OR temperature OR water stress OR climat* OR weather) AND (surviv* OR longevity OR lifespan OR life span OR life expectancy OR mortality))) all" [340 hits] [7 February, 2016]

Google Scholar: "Aedes humidity survival adult". Downloaded citations for first 200 hits (via Zotero) of about 9,620, sorted by relevance. [9 February, 2016]

LILACS, PAHO, CUMED, and MedCarib (all via http://pesquisa.bvsalud.org/): "tw:((aedes) AND ((humid* OR arid* OR 'vapor pressure' OR desiccat* OR precipitation OR temperature OR 'water stress' OR climat* OR weather) OR (humedad OR aridez OR 'presión de vapor' OR desecación OR precipitación OR temperatura OR 'estrés hídrico' OR clima)) AND ((surviv* OR longevity OR lifespan OR 'life span' OR 'life expectancy' OR mortality) OR (supervivencia OR longevidad OR mortalidad))) AND (instance:"regional") AND ( db:("LILACS" OR "CUMED" OR "PAHO" OR "MedCarib") AND type:("article" OR "project document" OR "monography" OR "congress and conference"))" with no language restrictions. [188 hits] [11 February, 2016]

WHOLIS: "Aedes survival" [0 hits] [12 February, 2016]

Scopus: "ALL (aedes AND humidity AND survival AND adult )" [340 hits] [13 February, 2016]

**Supplemental Results**

******

**Figure S1.1.** Flow diagram for study selection. Many studies were excluded based on multiple criteria but are tabulated here under only one criterion by which they were deemed ineligible.

***
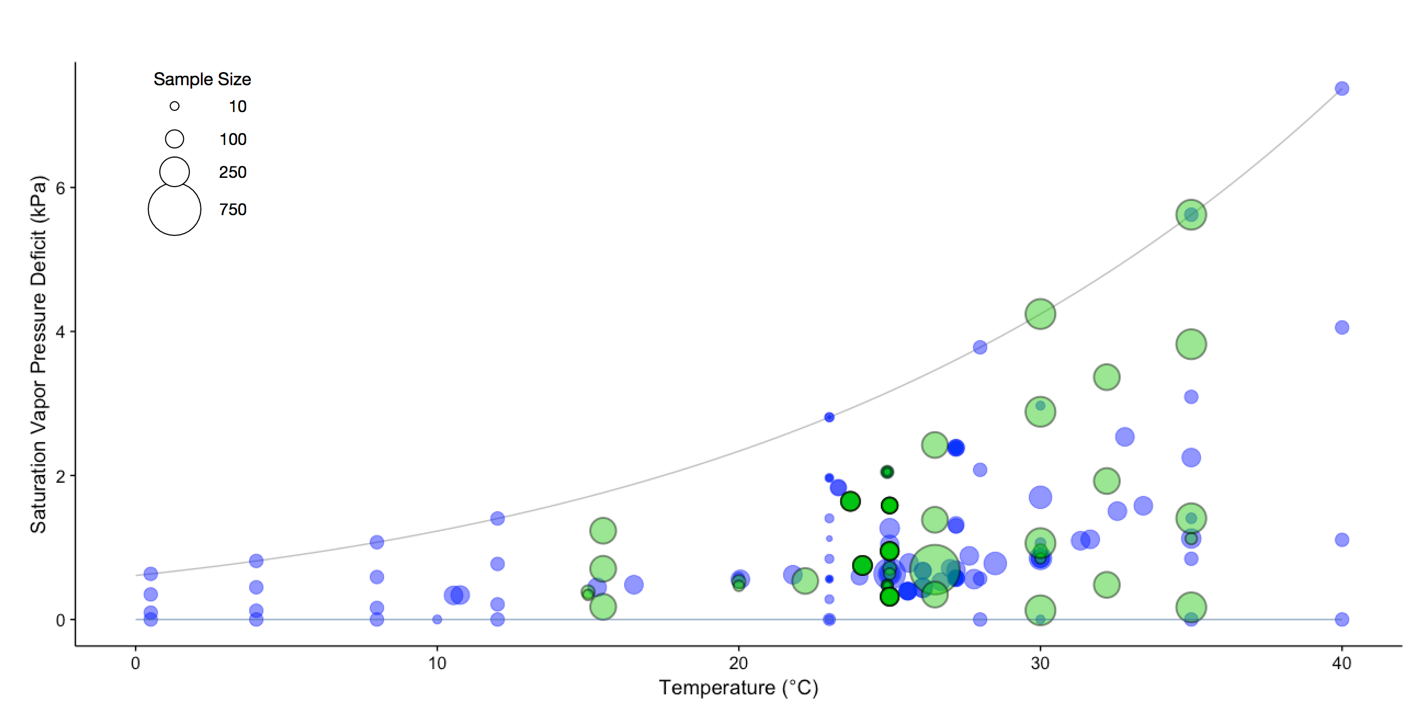
***

**Figure S1.2.** Distribution of mean temperature and saturation vapor pressure deficits employed by studies included in the present review. Circles represent individual experiments and are colored by species: *Aedes aegypti* (blue, without borders) or *Ae. albopictus* (green, with gray borders). Circle size is proportional to total sample size for an experiment. Lines represent 0% (upper line) and 100% (lower line) RH.


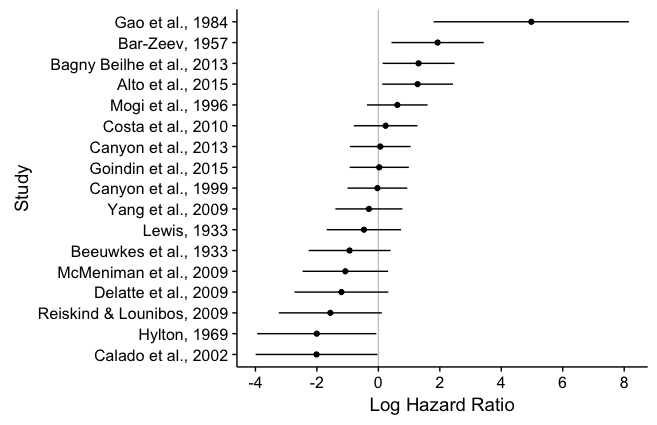


**Figure S1.3.** Study-level random effects from mixed effects Cox regression model for studies included in the present review. Estimates and 95% confidence intervals are pooled across all 500 simulated data sets and reflect study-specific differences in mortality hazards after conditioning on modelled covariates.

***
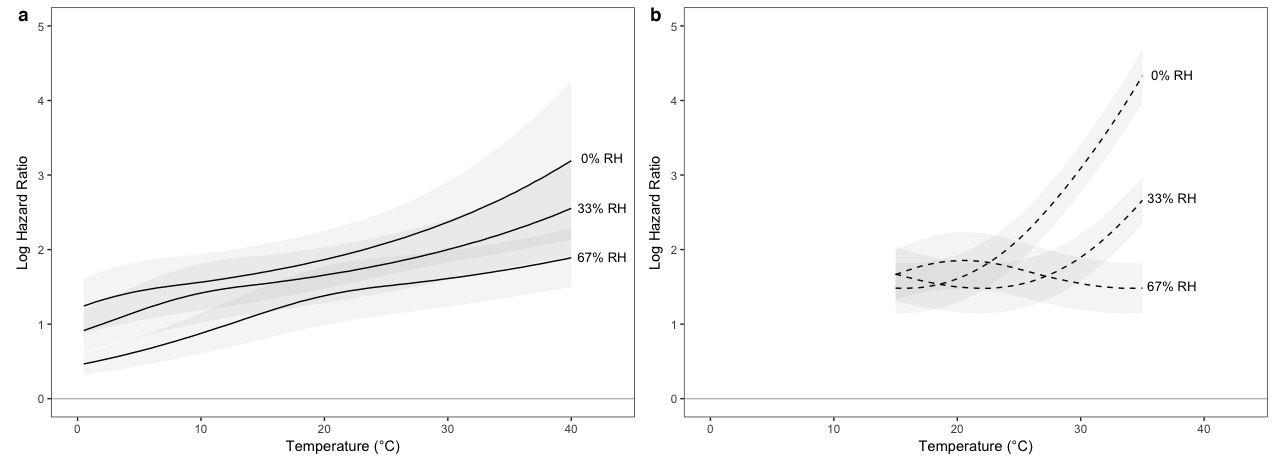
***

**Figure S1.4.** Pooled joint effects estimates (mean log hazard ratios, with 95% CI) from stratified model analysis for select relative humidities (%), relative to saturation (100% RH) at a given temperature, for (**a**) *Aedes aegypti* and (**b**) *Ae. albopictus*. Results reflect the temperature- and species-dependent associations of desiccation with mortality risk.

**Sources of Potential Error or Uncertainty**

**Table** **S1.1**. Ascertainment of potential sources of error or uncertainty in included studies.

| **Source** | **Highly Variable Temp./Humidity Within Single Experiments** | **Inconsistent Experimental Nutrition/ Hydration** | **Nutrition Provided Prior to Start of Experiments** | **Experiments Began >1 Day Post-Eclosion** | **Mating and Oviposition Prevented** | **Stock Colonies w/ Many Generations in Captivity** |
| --- | --- | --- | --- | --- | --- | --- |
| Alto et al., 2015 | No | No | No | No | Yes | No |
| Bagny Beilhe et al., 2013 | No | No | No | No | No | No |
| Bar-Zeev, 1957 | No | No | Unknown | Yes | Unknown | Unknown |
| Beeuwkes et al., 1933 | Yes | Yes | No | No | No | No |
| Calado and Navarro-Silva, 2002 | No | No | No | No | No | No |
| Canyon et al., 1999 | No | No | No | No | No | No |
| Canyon et al., 2013 | No | Yes | Yes | Yes | No | Unknown |
| Costa et al., 2010 | No | No | Yes | Unknown | No | Unknown |
| Delatte et al., 2009 | No | No | No | No | No | No |
| Gao et al., 1984 | No | No | Yes | Yes | No | Unknown |
| Goindin et al., 2015 | No | No | Yes | Yes | No | No |
| Hylton, 1969 | No | No | No | No | Yes | Unknown |
| Lewis, 1933 | No | Yes | No | Yes | Yes | Yes |
| McMeniman et al., 2009 | Yes (some experiments) | Yes | No | No | No | Yes |
| Mogi et al., 1996 | No | No | No | No | No | No |
| Reiskind and Lounibos, 2009 | No | No | Yes | Yes | Yes | No |
| Yang et al., 2009 | Yes | No | No | No | No | Unknown |

**Text 2. Additional Studies of Note**

Some studies did not meet the strict criteria for inclusion in our review but contain information of relevance to the present study. Lucio et al. ([1]; also Degallier et al. [2]) analyzed a set of survival data for adult *Ae. aegypti* from multiple sites in Fortaleza, Brazil, and found evidence of age-dependent mortality as well as significant associations between minimum or maximum SVPD and mortality risk at some sites, though the direction of the effect was mixed. Li et al. [3] compared survival of caged adult *Ae. albopictus* in urban, suburban and rural environments in two seasons, and found that longevity was greater in the cooler season (on average roughly 23 °C and 71% RH vs. 29 °C and 82% RH) and in the urban habitat; temperature and humidity profiles did not differ dramatically among locations and it is unclear whether experimental populations were identical. Lansdowne and Hacker [4] compared survival in five strains of *Ae. aegypti*, having widely differing geographic origins, under constant laboratory conditions versus fluctuating conditions in the external environment. Results suggested significant variation in mortality among strains but little effect of fluctuating versus constant conditions, in contrast with some studies [5-8]. We did not include this paper in our analysis because it is unclear which temperature and humidity values the outdoor populations experienced. Finally, Machado-Allison and Craig [9] examined desiccation tolerance in 15 geographically diverse *Ae. aegypti* populations, and although urban (*Ae. aegypti aegypti*) populations demonstrated higher survival under desiccating conditions than sylvan (*Ae. aegypti formosus*) populations, longevity did not differ significantly among populations within each subspecies, even between those from arid versus wetter environments.

1. Lucio PS, Degallier N, Servain J, Hannart A, Durand B, Souza RND, Ribeiro ZM. A case study of the influence of local weather on *Aedes aegypti* (L.) aging and mortality. *J Vector Ecol*. 2013;38(1),20-37.
2. Degallier N, Servain J, Lucio PS, Hannart A, Durand B, de Souza RN, Ribeiro ZM. The influence of local environment on the aging and mortality of *Aedes aegypti* (L.): case study in Fortaleza-CE, Brazil. *J Vector Ecol*. 2012;37,428–41.
3. Li Y, Kamara F, Zhou G, Puthiyakunnon S, Li C, Liu Y, et al. Urbanization increases *Aedes albopictus* larval habitats and accelerates mosquito development and survivorship. *PLOS Negl Trop Dis*. 2014;8,e3301.
4. Lansdowne C, Hacker CS. The effect of fluctuating temperature and humidity on the adult life table characteristics of five strains of *Aedes aegypti*. *J Med Ent*. 1975;11,723–33.
5. Lambrechts L, Paaijmans KP, Fansiri T, Carrington LB, Kramer LD, Thomas MB, Scott TW. Impact of daily temperature fluctuations on dengue virus transmission by *Aedes aegypti*. *Proc Nat Acad USA*. 2011;108, 7460–7465.
6. Carrington LB, Armijos MV, Lambrechts L, Barker CM, Scott TW. Effects of fluctuating daily temperatures at critical thermal extremes on *Aedes aegypti* life-history traits. *PLOS One*. 2013a;8,e58824.
7. Carrington LB, Armijos MV, Lambrechts L, Scott TW. Fluctuations at a low mean temperature accelerate dengue virus transmission by *Aedes aegypti*. *PLOS Negl Trop Dis*. 2013b;7.
8. Carrington LB, Seifert SN, Willits NH, Lambrechts L, Scott TW. Large diurnal temperature fluctuations negatively influence *Aedes aegypti* (Diptera: Culicidae) life-history traits. *J Med Ent*. 2013c;50,43–51.
9. Machado-Allison CE, Craig GB. Geographic variation in resistance to desiccation in *Aedes aegypti* and *A. atropalpus* (Diptera: Culicidae). *Annals Ent Soc America*. 1972;65(3),542–547.
