# Supplementary material for: Effects of desiccation stress on adult female longevity in Aedes aegypti and Ae. albopictus (Diptera: Culicidae): results of a systematic review and pooled survival analysis
Source: Parasit Vectors. 2018 Apr 25;11:267. doi: 10.1186/s13071-018-2808-6 (PMC5918765; doi:10.1186/s13071-018-2808-6)
Supplement: Supplementary file 3 — Model specification of the full dual-species Cox regression model, with pooled coefficient estimates. (PDF 103 kb) [file 13071_2018_2808_MOESM3_ESM.pdf]

Cox Regression Model Specification

The formula below gives the specification for the full dual-species Cox regression model. Model coefficients represent mean values over 500 simulated data sets.

$$\text{Prob}\{T \geq t \mid \text{article}_\text{i}\text{d} = i\} = S_i(t)^{e^{X\beta}}, \quad \text{where}$$

$X\hat{\beta} =$

$$\begin{aligned}
&3.418483 \\
&- 0.07404618\ \text{temperature} - 0.0001210023(\text{temperature} - 10.76)_+^3 \\
&+ 0.009569496\ (\text{temperature} - 25)_+^3 \\
&- 0.01189255\ (\text{temperature} - 27.2)_+^3 \\
&+ 0.002444053\ (\text{temperature} - 35)_+^3 + 1.388051[\text{albopictus}] \\
&+ 2.231304\ \text{SVPD} - 1.301778\ (\text{SVPD} - 0.127279)_+^3 \\
&+ 2.670574\ (\text{SVPD} - 0.633498)_+^3 \\
&- 1.368082\ (\text{SVPD} - 1.11352)_+^3 \\
&- 0.0007133561\ (\text{SVPD} - 3.823)_+^3 \\
&- 1.796275\ \text{water} - 3.186897\ \text{sugar} - 2.0946\ \text{blood} \\
&+ [\text{albopictus}][-0.105522\ \text{temperature} + 0.0006491691(\text{temperature} - 10.76)_+^3 \\
&- 0.01416306\ (\text{temperature} - 25)_+^3 \\
&+ 0.01614036\ (\text{temperature} - 27.2)_+^3 \\
&- 0.002626462(\text{temperature} - 35)_+^3] \\
&+ [\text{albopictus}][1.148352\ \text{SVPD} \\
&- 1.589431\ (\text{SVPD} - 0.127279)_+^3 \\
&+ 3.67276\ (\text{SVPD} - 0.633498)_+^3 \\
&- 2.155463\ (\text{SVPD} - 1.11352)_+^3 \\
&+ 0.07213333\ (\text{SVPD} - 3.823)_+^3] \\
&+ 2.190672\ \text{sugar} \times \text{blood}
\end{aligned}$$

and $[c] = 1$ if subject is in group c, 0 otherwise; $(x)_+ = x$ if $x > 0$, 0 otherwise.

temperature: Temperature ($^\circ$C)
SVPD: Saturation vapor pressure deficit (kPa)
albopictus: Indicator for species (*Aedes albopictus* = 1)
water: Indicator for water provision (water provided = 1)
sugar: Indicator for sugar provision (sugar provided = 1)
blood: Indicator for blood provision (blood provided = 1)
