# Supplementary material for: Effects of desiccation stress on adult female longevity in Aedes aegypti and Ae. albopictus (Diptera: Culicidae): results of a systematic review and pooled survival analysis
Source: Parasit Vectors. 2018 Apr 25;11:267. doi: 10.1186/s13071-018-2808-6 (PMC5918765; doi:10.1186/s13071-018-2808-6)
Supplement: Supplementary file 4 — Table S4.1. Single-study models used for sensitivity analyses assessing consistency of pooled model results with individual studies. Models represent the most complex treatment of variables in the pooled model possible for a given study. Figure S4.1. Comparison of model estimates for pooled (black) and single-study (blue) analyses of log hazard ratios (with 95% CI) by temperature (°C) for Aedes aegypti. Pooled model estimates are relative to a reference of Ae. aegypti at 27.5 °C. Figure S4.2. Comparison of model estimates for pooled (black) and single-study (blue) analyses of log hazard ratios (with 95% CI) by temperature (°C) for Aedes albopictus. Figure S4.3. Comparison of model estimates for pooled (black) and single-study (blue) analyses of log hazard ratios (with 95% CI) by saturation vapor pressure deficit (kPa) for Aedes aegypti. Pooled model estimates are relative to a reference of Ae. aegypti at full saturation. Figure S4.4. Comparison of model estimates for pooled (black) and single-study (blue) analyses of log hazard ratios (with 95% CI) by saturation vapor pressure deficit (kPa) for Aedes albopictus. Figure S4.5. Comparison of model estimates from all studies (black) and with individual studies excluded (blue) of log hazard ratios (with 95% CI) by temperature (°C) for Aedes aegypti (solid lines) and Ae. albopictus (dashed lines). Model estimates are relative to a reference of Ae. aegypti at 27.5 °C. Figure S4.6. Comparison of model estimates from all studies (black) and with individual studies excluded (blue) of log hazard ratios (with 95% CI) by saturation vapor pressure deficit (kPa) for Aedes aegypti (solid lines) and Ae. albopictus (dashed lines). Figure S4.7. Comparison of model estimates after simulation under mixed Weibull and log-logistic survival time distributions (black, bold lines) and under the Weibull distribution alone (blue, thin lines) for (a) temperature and (b) saturation vapor pressure deficit. Estimates are shown for Aedes aegypti (s [file 13071_2018_2808_MOESM4_ESM.docx]

**Additional file 4**

**Sensitivity Analysis Results**

**Table S4.1.** Single-study models used for sensitivity analyses assessing consistency of pooled model results with individual studies. Models represent the most complex treatment of variables in the pooled model possible for a given study. In some studies, temperature and SVPD could not be modeled simultaneously due to complete collinearity and were evaluated with separate models.

| Source | Single-Study Models^a^ |
| --- | --- |
| Alto et al., 2015 | SVPD*species |
| Bagny Beilhe et al., 2013 | (1) SVPD^3^  (2) temp^3^ |
| Bar-Zeev, 1957 | rcs(temp, 4) + rcs(SVPD, 4) |
| Beeuwkes et al., 1933 | rcs(temp, 4) + rcs(SVPD, 4) + blood |
| Calado et al., 2002 | (1) SVPD^2^  (2) temp^2^ |
| Canyon et al., 1999 | SVPD |
| Canyon et al., 2013 | SVPD + sugar*blood |
| Costa et al., 2010 | temp^2^ + rcs(SVPD, 3) |
| Delatte et al., 2009 | (1) SVPD^3^  (2) temp^3^ |
| Gao et al., 1984 | temp + rcs(SVPD, 4) |
| Goindin et al., 2015 | (1) SVPD^2^  (2) temp^2^ |
| Hylton, 1969 | temp^3^ + rcs(SVPD, 4) |
| Lewis, 1933 | temp^2^ + rcs(SVPD, 4) |
| McMeniman et al., 2009 | (1) SVPD + blood  (2) temp + blood |
| Mogi et al., 1996 | SVPD*species + water |
| Reiskind and Lounibos, 2009 | SVPD*species |
| Yang et al., 2009 | (1) rcs(SVPD, 4)  (2) rcs(temp, 4) |
| ^a^temp: temperature (°C); blood: access to bloodmeals provided; sugar: access to sugar sources provided; water: access to water provided; rcs: restricted cubic spline; SVPD: saturation vapor pressure deficit (kPa). (*) indicates a modeled interaction. | |


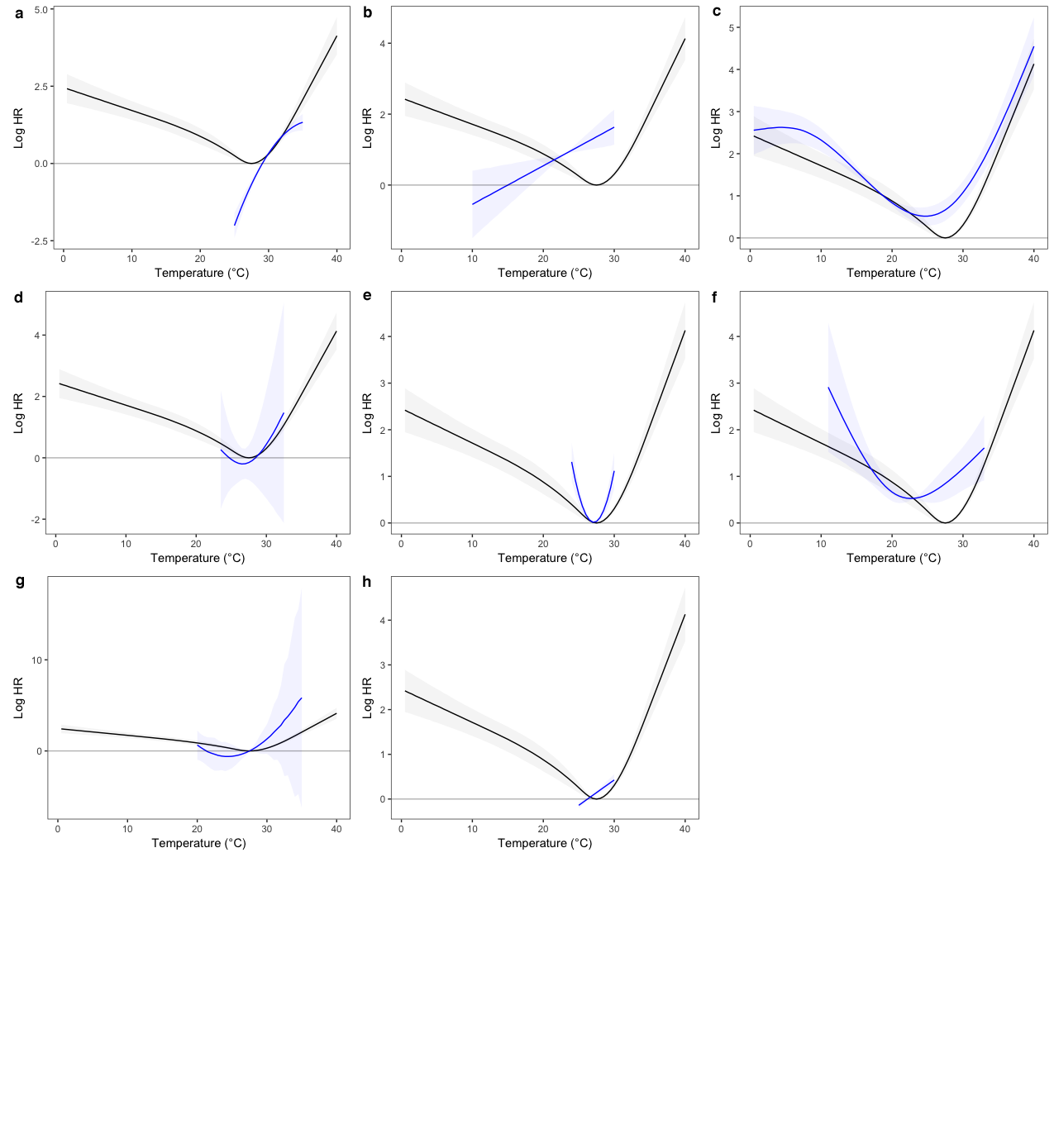


**Figure S4.1.** Comparison of model estimates for pooled (black) and single-study (blue) analyses of log hazard ratios (with 95% CI) by temperature (°C) for *Aedes aegypti*. Pooled model estimates are relative to a reference of *Ae. aegypti* at 27.5 °C. Single-study estimates are mapped to pooled model estimates at the mean temperature value for each study and are restricted to the range of values present in the data for each study. Only studies for which temperature constrasts could be evaluated are shown. (**a**) Costa et al., 2010; (**b**) Lewis, 1933; (**c**) Bar-Zeev, 1957; (**d**) Beeuwkes et al., 1933; (**e**) Goindin et al., 2015; (**f**) Yang et al., 2009; (**g**) Bagny Beilhe et al., 2013; (**h**) McMeniman et al., 2009.

**
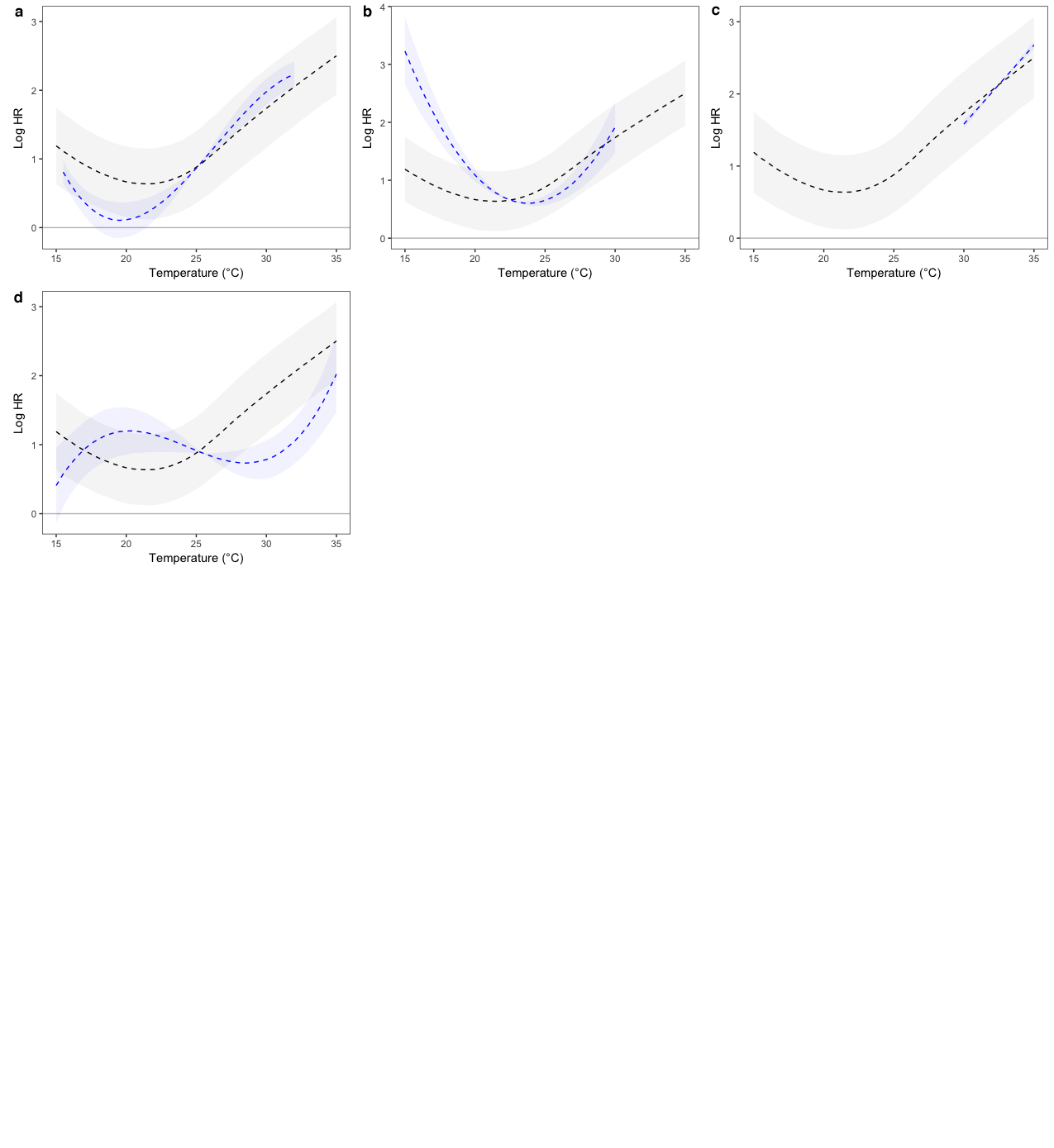
**

**Figure S4.2.** Comparison of model estimates for pooled (black) and single-study (blue) analyses of log hazard ratios (with 95% CI) by temperature (°C) for *Aedes albopictus*. Pooled model estimates are relative to a reference of *Ae. aegypti* at 27.5 °C. Single-study estimates are mapped to pooled model estimates at the mean temperature value for each study and are restricted to the range of values present in the data for each study. Only studies for which temperature constrasts could be evaluated are shown. (**a**) Hylton, 1969; (**b**) Calado and Navarro-Silva, 2002; (**c**) Gao et al., 1984; (**d**) Delatte et al., 2009.


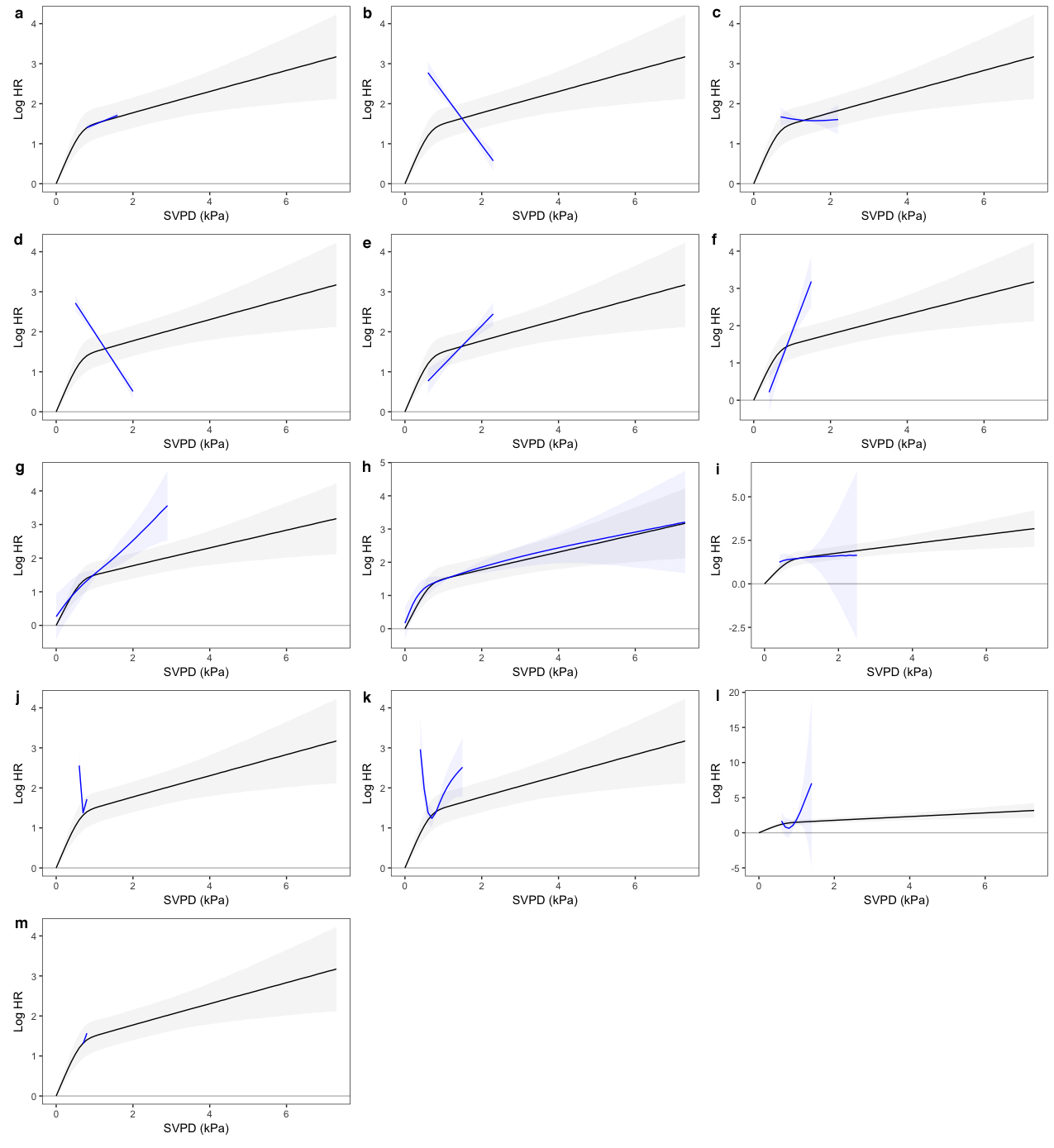


**Figure S4.3.** Comparison of model estimates for pooled (black) and single-study (blue) analyses of log hazard ratios (with 95% CI) by saturation vapor pressure deficit (kPa) for *Aedes aegypti*. Pooled model estimates are relative to a reference of *Ae. aegypti* at full saturation. Single-study estimates are mapped to pooled model estimates at the mean SVPD value for each study and are restricted to the range of values present in the data for each study. Only studies for which SVPD contrasts could be evaluated are shown. (**a**) Alto et al., 2015; (**b**) Canyon et al., 2013; (**c**) Costa et al., 2010; (**d**) Reiskind and Lounibos, 2009; (**e**) Canyon et al., 1999; (**f**) Mogi et al., 1996; (**g**) Lewis, 1933; (**h**) Bar-Zeev, 1957; (**i**) Beeuwkes et al., 1933; (**j**) Goindin et al., 2015; (**k**) Yang et al., 2009; (**l**) Bagny Beilhe et al., 2013; (**m**) McMeniman et al., 2009.

**
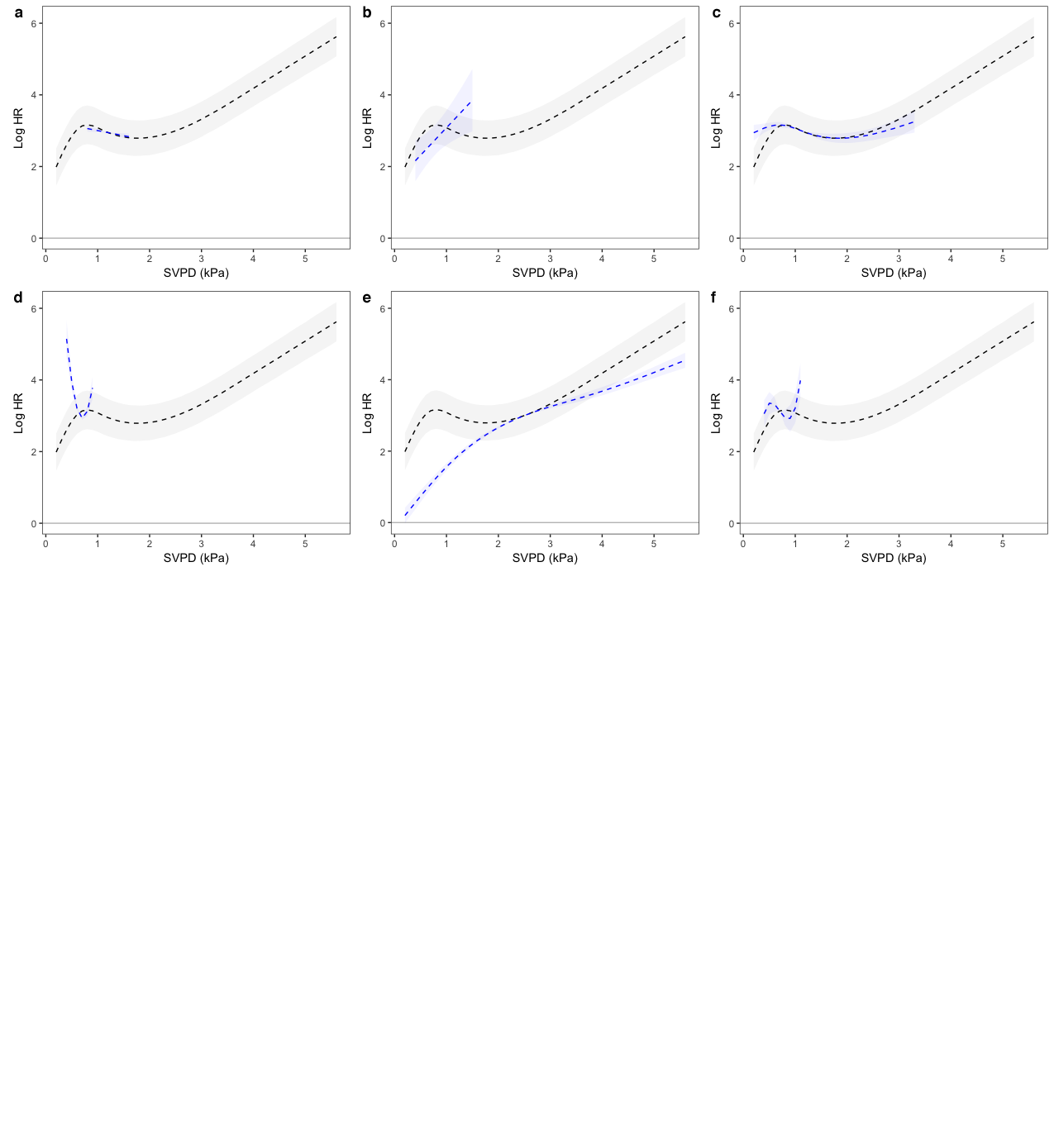
**

**Figure S4.4.** Comparison of model estimates for pooled (black) and single-study (blue) analyses of log hazard ratios (with 95% CI) by saturation vapor pressure deficit (kPa) for *Aedes albopictus*. Pooled model estimates are relative to a reference of *Ae. aegypti* at full saturation. Single-study estimates are mapped to pooled model estimates at the mean SVPD value for each study and are restricted to the range of values present in the data for each study. Only studies for which SVPD contrasts could be evaluated are shown. (**a**) Alto et al., 2015; (**b**) Mogi et al., 1996; (**c**) Hylton, 1969; (**d**) Calado and Navarro-Silva, 2002; (**e**) Gao et al., 1984; (**f**) Delatte et al., 2009.


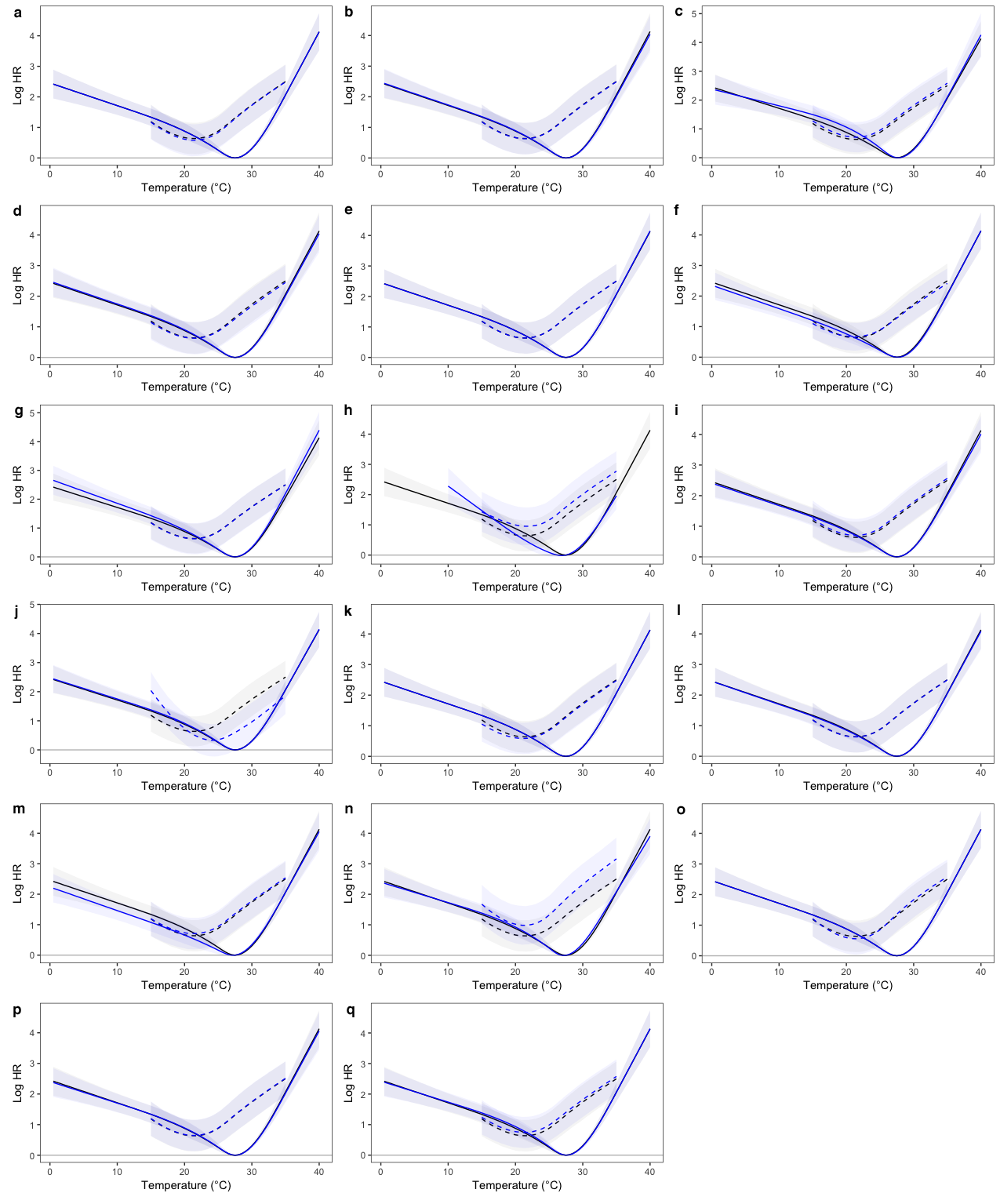


**Figure S4.5.** Comparison of model estimates from all studies (black) and with individual studies excluded (blue) of log hazard ratios (with 95% CI) by temperature (°C) for *Aedes aegypti* (solid lines) and *Ae. albopictus* (dashed lines). Model estimates are relative to a reference of *Ae. aegypti* at 27.5 °C and are restricted to the range of values present in the data for each species. (**a**) Alto et al., 2015; (**b**) Canyon et al., 2013; (**c**) Costa et al., 2010; (**d**) Reiskind and Lounibos, 2009; (**e**) Canyon et al., 1999; (**f**) Mogi et al., 1996; (**g**) Lewis, 1933; (**h**) Bar-Zeev, 1957; (**i**) Beeuwkes et al., 1933; (**j**) Hylton, 1969; (**k**) Calado and Navarro-Silva, 2002; (**l**) Goindin et al., 2015; (**m**) Yang et al., 2009; (**n**) Gao et al., 1984; (**o**) Delatte et al., 2009; (**p**) Bagny Beilhe et al., 2013; (**q**) McMeniman et al., 2009.

**
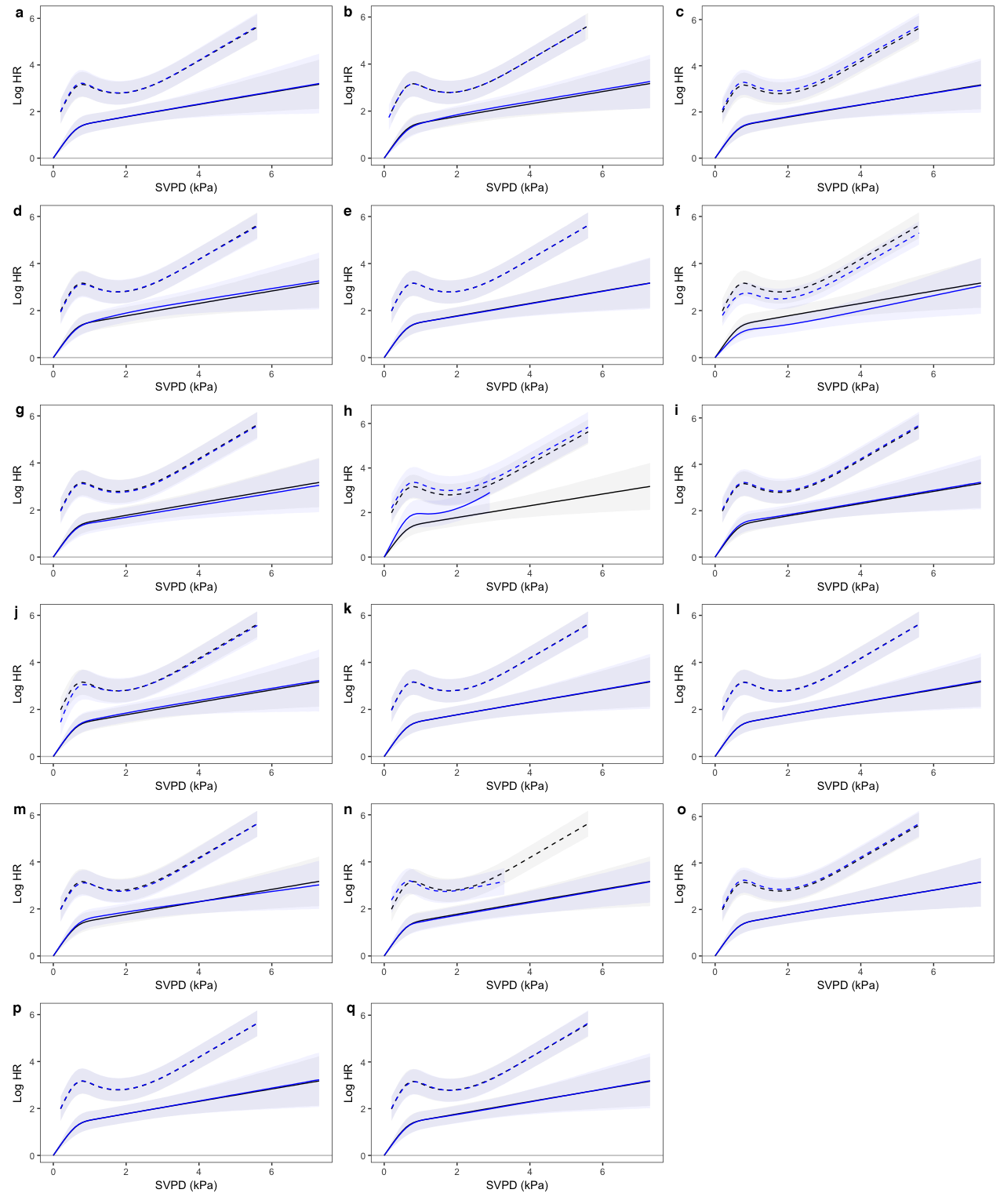
**

**Figure S4.6.** Comparison of model estimates from all studies (black) and with individual studies excluded (blue) of log hazard ratios (with 95% CI) by saturation vapor pressure deficit (kPa) for *Aedes aegypti* (solid lines) and *Ae. albopictus* (dashed lines). Model estimates are relative to a reference of *Ae. aegypti* at full saturation and are restricted to the range of values present in the data for each species. (**a**) Alto et al., 2015; (**b**) Canyon et al., 2013; (**c**) Costa et al., 2010; (**d**) Reiskind and Lounibos, 2009; (**e**) Canyon et al., 1999; (**f**) Mogi et al., 1996; (**g**) Lewis, 1933; (**h**) Bar-Zeev, 1957; (**i**) Beeuwkes et al., 1933; (**j**) Hylton, 1969; (**k**) Calado and Navarro-Silva, 2002; (**l**) Goindin et al., 2015; (**m**) Yang et al., 2009; (**n**) Gao et al., 1984; (**o**) Delatte et al., 2009; (**p**) Bagny Beilhe et al., 2013; (**q**) McMeniman et al., 2009.

**
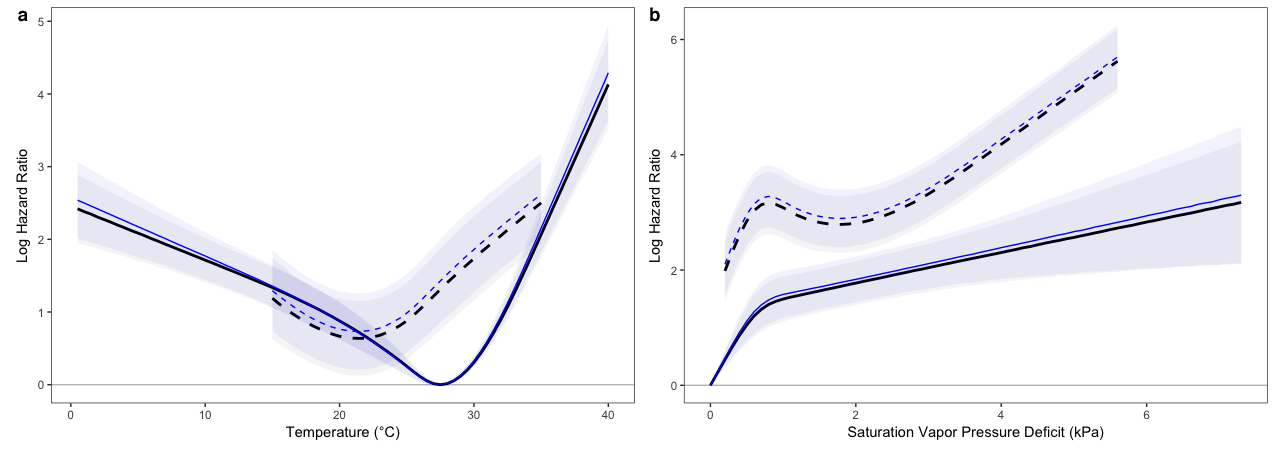
Figure S4.7.** Comparison of model estimates after simulation under mixed Weibull and log-logistic survival time distributions (black, bold lines) and under the Weibull distribution alone (blue, thin lines) for (**a**) temperature and (**b**) saturation vapor pressure deficit. Estimates are shown for *Aedes aegypti* (solid lines) and *Ae. albopictus* (dashed lines), and are relative to a reference of *Ae. aegypti* at 27.5 °C (A) or *Ae. aegypti* at full saturation (B). Estimates are restricted to the range of values present in the data for each species.

**Text 1. Relative Mortality Hazards Between *Ae. aegypti* and *Ae. albopictus***

Our analyses included experimental data from three studies [1-3] that evaluated survival in both *Ae. aegypti* and *Ae. albopictus*, enabling estimation of relative mortality hazards between these species. All three studies estimated greater longevity in *Ae. aegypti* than *Ae. albopictus* under most experimental conditions. Contrary results were obtained by the experiments of Bagny Beilhe et al. [4] and Delatte et al. [5], which were undertaken with populations of *Ae. aegypti* and *Ae. albopictus* (respectively) from Réunion and which estimated greater average longevities for *Ae. albopictus* than *Ae. aegypti*. Given that these studies were conducted at different times and by mostly different research teams, we treated them as separate strata in our stratified survival analysis. A reanalysis of our model with these studies combined into one stratum reduced but did not reverse the relative survival advantage of *Ae. aegypti* over *Ae. albopictus* (Figure S8).

**
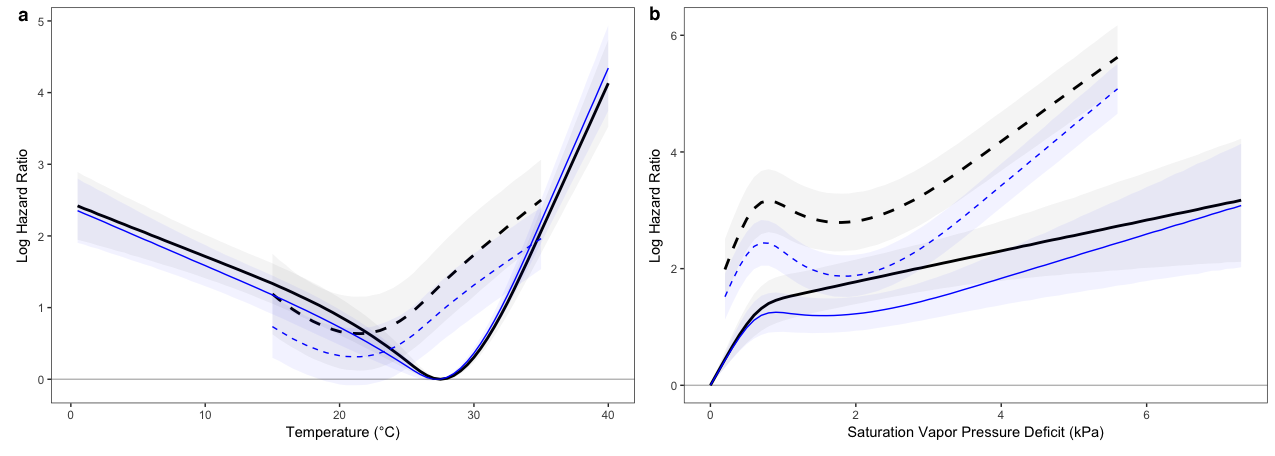
**

**Figure S4.8.** Comparison of model estimates with (blue, thin lines) and without (black, bold lines) treatment of Bagny Beilhe et al. [4] and Delatte et al. [5] as a single stratum in the Cox regression analysis, for (**a**) temperature and (**b**) saturation vapor pressure deficit. Estimates are shown for *Aedes aegypti* (solid lines) and *Ae. albopictus* (dashed lines), and are relative to a reference of *Ae. aegypti* at 27.5 °C (A) or *Ae. aegypti* at full saturation (B). Estimates are restricted to the range of values present in the data for each species.

1. Alto BW, Bettinardi DJ, Ortiz S. Interspecific larval competition differentially impacts adult survival in dengue vectors. *J Med Ent*. 2015;52(2):163–170.
2. Mogi M, Miyagi I, Abadi K, Syafruddin. Inter- and intra-specific variation in resistance to desiccation by adult *Aedes* (*Stegomyia*) spp. (Diptera: Culicidae) from Indonesia. *J Med Ent.* 1996;33:53–57.
3. Reiskind MH, Lounibos LP. Effects of intraspecific larval competition on adult longevity in the mosquitoes *Aedes aegypti* and *Aedes albopictus*. *Med Vet Ent.* 2009;23(1):62–68.
4. Bagny Beilhe L, Delatte H, Juliano SA, Fontenille D, Quilici S. Ecological interactions in *Aedes* species on Reunion Island. *Med Vet Ent*. 2013;27:387-397.
5. Delatte H, Gimonneau G, Triboire A, Fontenille D. Influence of temperature on immature development, survival, longevity, fecundity, and gonotrophic cycles of *Aedes albopictus*, vector of chikungunya and dengue in the Indian Ocean. *J Med Ent.* 2009;46:33-41.
